# Colour Helmholtz Stereopsis for Reconstruction of Dynamic Scenes with Arbitrary Unknown Reflectance

**DOI:** 10.1007/s11263-016-0951-0

**Published:** 2016-09-20

**Authors:** Nadejda Roubtsova, Jean-Yves Guillemaut

**Affiliations:** Centre for Vision, Speech and Signal Processing, University of Surrey, Guildford, GU2 7XH UK

**Keywords:** 3D reconstruction, Dynamic scenes, Arbitrary BRDF, Helmholtz Stereopsis

## Abstract

Helmholtz Stereopsis is a powerful technique for reconstruction of scenes with arbitrary reflectance properties. However, previous formulations have been limited to static objects due to the requirement to sequentially capture reciprocal image pairs (i.e. two images with the camera and light source positions mutually interchanged). In this paper, we propose colour Helmholtz Stereopsis—a novel framework for Helmholtz Stereopsis based on wavelength multiplexing. To address the new set of challenges introduced by multispectral data acquisition, the proposed Colour Helmholtz Stereopsis pipeline uniquely combines a tailored photometric calibration for multiple camera/light source pairs, a novel procedure for spatio-temporal surface chromaticity calibration and a state-of-the-art Bayesian formulation necessary for accurate reconstruction from a minimal number of reciprocal pairs. In this framework, reflectance is spatially unconstrained both in terms of its chromaticity and the directional component dependent on the illumination incidence and viewing angles. The proposed approach for the first time enables modelling of dynamic scenes with arbitrary unknown and spatially varying reflectance using a practical acquisition set-up consisting of a small number of cameras and light sources. Experimental results demonstrate the accuracy and flexibility of the technique on a variety of static and dynamic scenes with arbitrary unknown BRDF and chromaticity ranging from uniform to arbitrary and spatially varying.

**Electronic supplementary material:**

The online version of this article (doi:10.1007/s11263-016-0951-0) contains supplementary material, which is available to authorized users.

## Introduction

3D reconstruction has been an active research area in computer vision in the past decades due to the high demand for it in numerous industrial applications. For example, modern heritage preservation projects set high standards for geometric accuracy on challenging data striving for sub-millimetre resolution accuracy and impeccable global shape. Real objects often have unknown complex surface reflectance with a non-trivial (non-Lambertian) and possibly spatially varying model. There is also much interest in capturing dynamic often non-rigid deformation. This paper tackles the combined challenge of dynamic scene reconstruction with complex arbitrary spatially varying reflectance properties.

Shape-from-Silhouette (Baumgard 1974; Matusik et al. 2000; Lazebnik et al. 2007; Liang and Wong 2010) is a classical geometric technique that is independent of surface reflectance. However, the resolution of structural concavities in the visual hulls (Laurentini 1994) is poor compared to intensity-based methods. The well-established intensity-based methods for 3D geometry reconstruction such as binocular (Scharstein and Szeliski 2002) and multi-view (Seitz et al. 2006) conventional stereo as well as photometric stereo (Woodham 1989; Basri et al. 2007; Higo et al. 2010) have demonstrated remarkable sub-millimetre geometric accuracies on tailored data. The known limitation of both methods however is the inherent inability to deal with unknown surface reflectance. Conventional stereo requires Lambertian (purely diffuse) bi-directional reflectance distribution function (BRDF) being unable to establish feature matches where surface specularities occur. Photometric stereo on the other hand requires the a priori knowledge of the BRDF that must be acquired as pre-processing by a cumbersome and often insufficiently accurate method. There has been work focussing on combining the global accuracy of conventional stereo with the high frequency detail obtained from shading cues (Ahmed et al. 2008; Wu et al. 2011) or photometric stereo (Vlasic et al. 2009; Anderson et al. 2011) to increase modelling accuracy. However, the inherent limitations due to complex reflectance remain. To our knowledge, Helmholtz Stereopsis (HS) is the only technique in existence capable of accurately modelling surfaces with an arbitrary BRDF. The technique’s acquisition set-up, proposed in the seed paper by Zickler et al. (2002), features reciprocal image pairs characterised by the mutually interchanged camera and light source. The reciprocity at acquisition allows to formulate a depth constraint with the dependence on the BRDF factored out and hence an expanded range of applicability.

Standard HS has been shown to achieve excellent results for rigid scenes with complex a priori unknown reflectance. However, standard HS is not scalable to dynamic scenes since it does not permit simultaneous acquisition of the minimum of three reciprocal pairs due the performed swap of the camera and light source. In this paper, we propose Colour Helmholtz Stereopsis (CL HS) where wavelength multiplexing is used to enable simultaneous capture of reciprocal pairs. Signal separation is achieved by using three cameras and three coloured light sources and treating each camera channel as a separate image (Fig. 5). The novel approach permits instantaneous capture of three reciprocal pairs but it also introduces a new set of challenges. The challenges are the acute need for photometric calibration of capturing equipment, signal dependence on surface chromaticity and the ambiguity introduced by the drastically reduced number of reciprocal pairs per point.

We address all these challenges by developing a complete practical pipeline for CL HS. The pipeline includes a generalisation of the white light photometric calibration procedure from Jankó et al. (2004) to accommodate for chromatic characteristics of the cameras and multispectral light sources. As surface colour will affect inter-channel compatibility we propose a novel method for surface chromaticity estimation. Spatially varying chromaticity in dynamic scenes is addressed in a procedure for propagation of statically estimated characteristics throughout the sequence. Together the processes of chromaticity estimation and propagation define a procedure for spatio-temporal chromatic calibration of the surface and permit chromatically unconstrained dynamic scene reconstruction. The freedom of the directional behaviour of surface BRDF (i.e. unconstrained surface material) is inherently a given as the reflectance-independent HS constraint is used for reconstruction. Further, to cope with the reduced number of reciprocal pairs, we incorporate the state-of-the-art Bayesian HS formulation from Roubtsova and Guillemaut (2014a) into the pipeline. To the best of our knowledge, our CL HS pipeline is the first approach capable of reconstructing dynamic scenes with arbitrary spatially varying reflectance.

## Related Work

The dominance of intensity-based reconstruction methods in terms of accuracy and practicality is evident from their ubiquitous use. Their conventional weakness is the reliance on the knowledge of surface-specific BRDF to separate geometry from reflectance in the sampled intensity response. The original formulations of both conventional and photometric stereo assume Lambertian reflectance. There has been noteworthy work aiming to generalise both techniques to more realistic cases.

Jin and colleagues (Jin et al. 2003, 2005) explicitly model non-Lambertian behaviour of scenes for conventional stereo at the expense of increased computational complexity. They propose a novel model-to-image discrepancy measure for non-Lambertian surfaces based on the more generic Ward’s reflectance model (Ward 1992) and formulated via the radiance tensor containing multiview reflectance observations per surface patch. In Oxholm and Nishino (2014), instead of explicit reflectance modelling, the approach aims for joint global estimation of shape and reflectance properties from a set of multiview images. Although the idea of joint inference of unknown parameters (i.e. shape and reflectance) based on observable characteristics (i.e. intensity and the illumination conditions) is plausible, the two estimates will remain inherently linked and the shape cannot be expected to be more accurate than the reflectance estimate whose full complexity is difficult to model with a finite number of images. Although conceptually interesting, non-Lambertian multiview conventional stereo methods comparatively do not deliver particularly accurate or high resolution results.

The topic of unconstrained reflectance has attracted even more attention in photometric stereo as unlike conventional stereo the technique is not fundamentally limited by the Lambertian assumption. One early attempt was the so-called photometric stereo by example (Hertzmann and Seitz 2005). The idea is to find relationships between surface normals and observed reflectance behaviour by sampling reference objects of known geometry under different illumination. The test object’s material is considered a linear combination of reference materials. With the assumption not being fundamental, the work cannot claim to have universally solved reflectance model dependence. In a more principled manner, Vogiatzis and Hernández (2012) address the difficulty of non-Lambertian reflectance in photometric stereo by fitting a Phong model (Phong 1975) based on the outliers from the Lambertian model estimation procedure. For problem tractability, the assumptions of monochromaticity and (spatially and spectrally) constant specular model parameters are made. In the formulation with the more complex Phong reflectance model, the model’s invertibility issues are tackled using the mentioned assumptions. The formulation is innovatively non-Lambertian, but the scene reflectance model allowed by the method is far from arbitrary but in fact limited by the many assumptions made (Phong model, monochromaticity, constant specular components parameters etc.) in pursuit of tractability and well-posedness of the mathematical formulation of the photometric stereo problem.

The aforementioned generalisation of conventional and photometric stereo to specific more complex models (Ward, Phong etc.) is not as fundamentally principled as the techniques based on generic or at the very least very common properties of the entire BRDF class. Specifically, these methods exploit various symmetries in the BRDF behaviour. In the photometric stereo approach of Holroyd et al. (2008) common half vector symmetries are utilised. The symmetries extend to some anisotropic microfacet-based models (Cook and Torrance 1982; Ashikmin et al. 2000; Ngan et al. 2005) but do not cover the entire class of physically valid BRDFs with the method having been found to fail in the face of retro-reflection and surfaces with asymmetric micro-geometries. Further, there has also been work (Alldrin and Zickler 2008; Zhou et al. 2013) on exploiting isotropy (i.e. rotation invariance about the normal) - a symmetry which, although not generic, is common for a majority of real-life reflectance models. The assumption of isotropy in photometric stereo allows the construction of the so-called iso-depth contours where all points are equidistant from the image plane. In Zhou et al. (2013), impressively accurate geometries of non-Lambertian surfaces are reconstructed by propagation of sparse surface points obtained by structure-from-motion along the constructed iso-depth contours. Relying on isotropic reflectance to build continuous iso-depth contours, these methods will also be sensitive to cast shadows and inter-reflections, not to mention the obvious requirement of an isotropic, although otherwise arbitrary, BRDF. In Tan et al. (2011), in addition to the wide-spread isotropy property of the BRDF, its generic symmetry of reciprocity is employed in the context of calibrated and uncalibrated photometric stereo. Although clearly a step towards generalisation to a wider class of BRDFs, the reliance of the proposed system on non-generic isotropy through its joint constraints with reciprocity still imposes limitations on the type of reflectance. To the best of our knowledge, no photometric stereo algorithm based solely on generic symmetries of the BRDF has ever been proposed.

In contrast, the generic reciprocity is the sole core underlying symmetry in an independent reconstruction technique of Helmholtz Stereopsis (HS). The use of the reciprocity symmetry exclusively makes HS the only intensity-based technique fundamentally independent of the BRDF. The subsequent development of HS, after its introduction by Zickler et al. (2002), included work on extensions for wider applicability and increased geometric accuracy. Guillemaut et al. (2004) propose modifications for accurate geometric reconstruction of highly textured surfaces by HS. In Guillemaut et al. (2008), a more physically meaningful HS constraint resulting in a Maximum Likelihood (ML) surface is formulated. In our recent work (Roubtsova and Guillemaut 2014a, 2015) Bayesian formulation of HS with a tailored prior jointly optimising depth and normal information is shown to produce superior results to the original ML formulation in Zickler et al. (2002). Variational approaches optimising over the entire surface (Delaunoy et al. 2010; Weinmann et al. 2012) have been proposed in order to extend HS to full 3D reconstruction. In Weinmann et al. (2012) HS is aided by a structured light technique to identify a consistent point set defining the reconstruction volume.

A major limitation of HS has always been its controlled set-up and the slow acquisition speed. Some of the impracticalities preventing the reconstruction technique from gaining wider popularity are addressed by the HS inventors in the follow-up papers. Firstly, Zickler et al. (2003) propose a binocular variant of HS where geometry is reconstructed from a single reciprocal pair by a differential approach. A partial differential equation of depth as a function of surface coordinates with prior initialisation produces a family of solutions, the ambiguity of which is resolved through a multi-pass optimisation. Although an excellent example of applied optimisation, binocular HS simplifies HS acquisition at the cost of vastly increased computational complexity and introduced reconstruction ambiguities. Another paper by Zickler (2006) addresses automatic online geometric calibration of a HS set-up using stable regions of interest: the texture-based and the inherent to HS specularity-based features. The paper proposes a method to avoid pre-calibration of the set-up but does not deal with the bottleneck issue of tedious sequential image acquisition limiting the scope of the technique to static scenes.

The same paper also touches upon automatic radiometric calibration of HS set-up using the inherent specularity-based features. In Zickler (2006), the definition of radiometric calibration is limited to measuring relative intensities of isotropic light sources. Provided the assumptions of equal camera responses and no spatial source intensity variation hold, such limited calibration is sufficient. A more general radiometric set-up calibration for HS, which does not rely on these assumptions, was proposed by Jankó et al. (2004). Using a sequence of localised calibration planes Jankó et al. calibrate for a spatially varying joint parameter describing sensitivity and radiance of a collocated camera and light source pair.

Unlike Zickler et al. (2003) who for HS simplification modify the reconstruction algorithm only, we propose a complete novel pipeline tailored for processing HS input for the first time acquired using wavelength multiplexing. Simultaneous multi-channel acquisition for dynamic scene reconstruction is known from the well-established Colour Photometric Stereo (CL PS). In Hernández et al. (2007) and its later extension Brostow et al. (2011), CL PS is shown to produce impressive reconstructions of dynamic scenes with untextured (uniform albedo) objects, specifically cloth deformation and facial expression sequences. By enforcing spatio-temporal smoothness, Jankó et al. (2010) extend the technique to textured surfaces, hence allowing spatial reflectance non-uniformity due to its chromaticity component. Both works however are limited to a Lambertian reflectance at each surface point, regardless whether chromaticity is uniform or allowed to be spatially varying across the surface. The applicability of the extension of CL PS to the non-Lambertian case from Vogiatzis and Hernández (2012) is limited because it relies on the accuracy of the data-driven fitting of a specular model whose variability is heavily constrained for mathematically tractability.

In contrast to the discussed state-of-the-art Colour Photometric Stereo techniques, the proposed Colour Helmholtz Stereopsis (CL HS) is valid for any arbitrary spatially varying BRDF. In this paper, we decouple the chromaticity component of the BRDF from the component dependent only on the illumination incidence and viewing angles (henceforth referred to as the directional component). As in standard HS, the directional component of BRDF in CL HS is made irrelevant by virtue of reciprocity at acquisition. In order to deal with the non-uniformity of the chromaticity component of the BRDF, we estimate chromaticity by integrating a novel calibration procedure into the pipeline. This builds upon our previous work (Roubtsova and Guillemaut 2014b, 2015) where static per-pixel surface chromaticity calibration permitted spatially varying chromaticity in static single-shot reconstructions only and dynamic scene reconstruction was limited to datasets with spatially uniform known chromaticity. The current work generalises the scope to dynamic scenes with spatially varying chromaticity by introducing an additional procedure for temporal propagation of surface chromaticity estimated in a reference frame. Spatio-temporal chromaticity calibration allows any arbitrary a priori unknown chromatic characteristics of the surface. Furthermore, likewise for inter-channel signal consistency at acquisition, in the proposed pipeline we generalise the previous work in photometric calibration of Jankó et al. (2004) to multiple multi-chromatic cameras and light sources. Hence by combining the unique properties of HS with a novel multi-spectral acquisition set-up and calibration procedures we obtain a method uniquely permitting dynamic scene reconstruction with fully arbitrary spatially varying BRDFs i.e. unconstrained in terms of both the directional and chromaticity components.

## White Light Helmholtz Stereopsis

Since this paper proposes a multi-spectral variant of Helmholtz Stereopsis (HS), as a reference we first of all introduce and formalise the theory of traditional White Light Helmholtz Stereopsis (WL HS) together with its calibration procedure from Jankó et al. (2004). Subsequently, we shall use the same formalisation framework to present our novel Colour Helmholtz Stereopsis (CL HS) formulation.

**Fig. 1:**
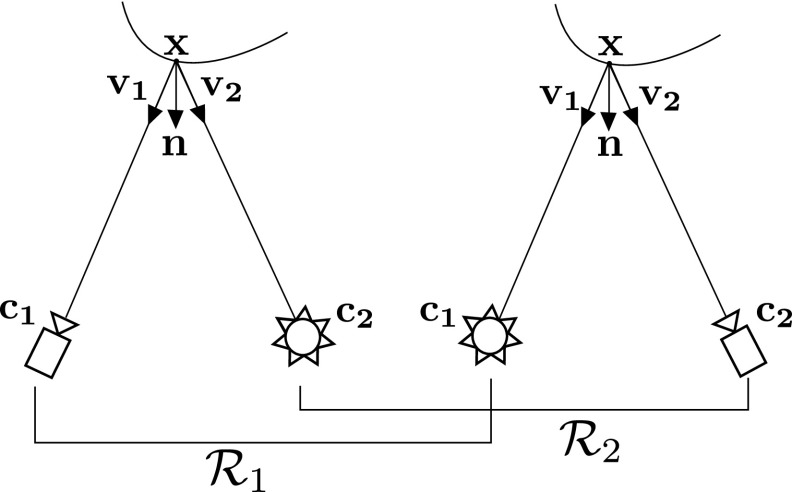
Reciprocal intensity sampling in White Light Helmholtz Stereopsis—virtual camera-light source collocation

To introduce HS, let us define a perspective camera $\mathcal {C}$ and a light source $\mathcal {S}$ centred at $\mathbf {c_{1}}$ and $\mathbf {c_{2}}$ respectively. In standard HS reciprocal image pairs are acquired with any $\mathcal {C}$ and $\mathcal {S}$ respectively first at locations $\mathbf {c_{1}}$ and $\mathbf {c_{2}}$ and then at $\mathbf {c_{2}}$ and $\mathbf {c_{1}}$ i.e. with the camera and light source mutually interchanged (Fig. 1). As in Jankó et al. (2004) we define the concept of Helmholtz camera $\mathcal {R}$ as a collocated camera and light source at some position $\mathbf {c}$. Traditionally, the collocation is virtual by either the camera/light source swap or by using a turntable to move the scene relative to the set-up (Fig. 1). Let us define virtually collocated $(\mathcal {C},\mathcal {S})$ pairs, $\mathcal {R}_{1} = (\mathcal {C}_{1},\mathcal {S}_{1})$ and $\mathcal {R}_{2}= (\mathcal {C}_{2},\mathcal {S}_{2})$ located at $\mathbf {c_{1}}$ and $\mathbf {c_{2}}$ respectively. The arrangement facilitates Helmholtz reciprocity as one image of the reciprocal pair is obtained with $\mathcal {C}_{1}$ and $\mathcal {S}_{2}$ at $\mathbf {c_{1}}$ and $\mathbf {c_{2}}$ and the other with $\mathcal {C}_{2}$ and $\mathcal {S}_{1}$ at $\mathbf {c_{2}}$ and $\mathbf {c_{1}}$ respectively. Note, in contrast to the state-of-the-art, the proposed method of CL HS presented in the next section will be based on physical collocation of $\mathcal {C}$ and $\mathcal {S}$ which means two distinct cameras and two light sources per pair of Helmholtz cameras (Fig. 4).

**Fig. 2:**
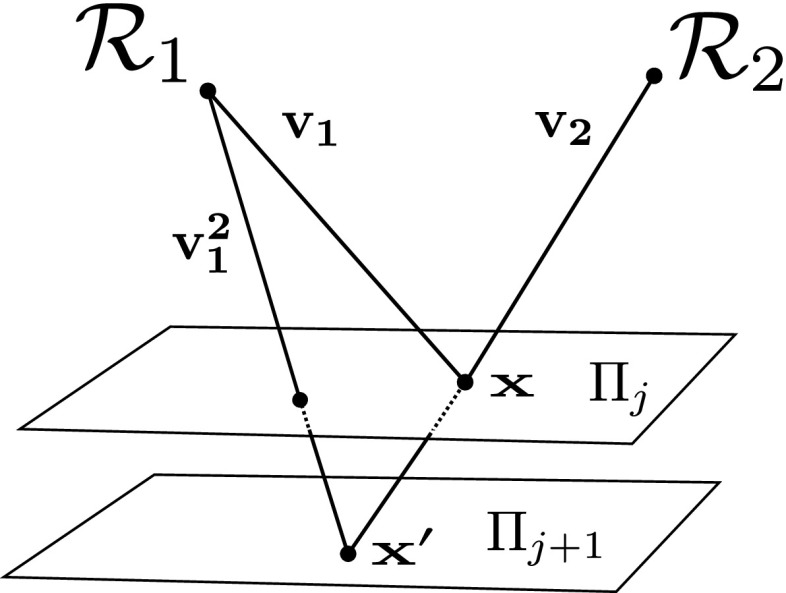
Geometry of the photometric calibration procedure by Jankó et al. for WL HS

Regardless whether it is realised through physical or virtual collocation, Helmholtz camera $\mathcal {R}$ is photometrically characterised by its radiance and sensitivity functions, $\rho$ and $\sigma$ respectively. With virtual collocation (i.e. a single pair of $\mathcal {C}$ and $\mathcal {S}$) the sensitivity distributions of $\mathcal {R}_1$ and $\mathcal {R}_2$ will be the same but the radiances may vary due to different light source orientations. With physical collocation the photometric distributions of Helmholtz cameras are uncorrelated. Both $\rho$ and $\sigma$ vary as a function of ray $\mathbf {v}$ from the surface point $\mathbf {x}$ to $\mathcal {R}$ (Fig. 2).

Hence, intensity $i_{1}$ at surface point $\mathbf {x}$ in the reciprocal pair image $I_{1}$ acquired with $\mathcal {R}_{2}$ as the light source and $\mathcal {R}_{1}$ as the camera can be expressed (Jankó et al. 2004) as:

1 $$\begin{aligned} i_{1} = \rho _{2}(\mathbf {v_{2}})\sigma _{1}(\mathbf {v_{1}})f_{r}(\mathbf {v_2},\mathbf {v_1}) \frac{ \mathbf {v_2} \cdot \mathbf {n} }{\Vert \mathbf {c_{2}} - \mathbf {x}\Vert ^2} \end{aligned}$$

where $\rho _{2}(\mathbf {v_{2}})$ is the radiance of $\mathcal {R}_{2}$ along $\mathbf {v_{2}}$ and $\sigma _{1}(\mathbf {v_{1}})$ is the sensor sensitivity of $\mathcal {R}_{1}$ along $\mathbf {v_{1}}$. Intensity $i_{2}$, which is the projection of $\mathbf {x}$ in the other reciprocal pair image, is obtained by interchanging the vector indices 1 and 2 in (1). Jankó et al. (2004) propose a method for photometric calibration of $\mathcal {R}$ in traditional WL HS. Specifically, for $\mathcal {R}_{1}$ and $\mathcal {R}_{2}$ as in Fig. 2 Jankó et al. (2004) calibrate for the radiance to sensitivity ratios:

2 $$\begin{aligned} \mu _k(\mathbf {v_{k}}) = \frac{\rho _{k}(\mathbf {v_{k}})}{{\sigma _{k}(\mathbf {v_{k}})}}, \;\; k=1,2 \end{aligned}$$

In WL HS the wavelength variable $\omega$ can be omitted from the BRDF expression $f_{r}(\mathbf {v_2},\mathbf {v_1})$ at a given surface point because of the constant spectral characteristics of sampling illumination and the camera sensor. Since the sampling and sampled frequencies are known to be constant, the reflection/absorption behaviour due to the chromaticity of the sampled point is consistent and the BRDF varies only with the local directional variables $\mathbf {v_1}$ and $\mathbf {v_2}$. The whiteness of the sampling spectral characteristics ensures a sufficient response for the widest range of surface colours. Reciprocal intensity measurements $i_{1}$ and $i_{2}$ can be combined into a single surface normal constraint eliminating the dependence on the BRDF $f_{r}(\mathbf {v_2},\mathbf {v_1})$ at that point. The elimination is based on Helmholtz reciprocity (Helmholtz 1925) - the invariance of optical behaviour in medium of a light ray and its reverse. For BRDF the implication first observed by Zickler et al. (2002) is that: $f_{r}(\mathbf{{v_1}},\mathbf{{v_2}}) = {f_{r}}(\mathbf {v_2},\mathbf {v_1})$. Via this equality reciprocal intensities $i_{1}$ and $i_{2}$ expressed as in (1) are linked, incorporating photometric calibration $\mu$, to give the normal constraint:

3 $$\begin{aligned} \left( \frac{\mu _{1}(\mathbf {v_1}) i_{1} }{\Vert \mathbf {c_{1}} - \mathbf {x}\Vert ^2} \mathbf {v_1} - \frac{\mu _{2}(\mathbf {v_2}) i_{2} }{\Vert \mathbf {c_{2}} - \mathbf {x}\Vert ^2} \mathbf {v_2}\right) \cdot \mathbf {n}= 0. \end{aligned}$$

**Fig. 3:**
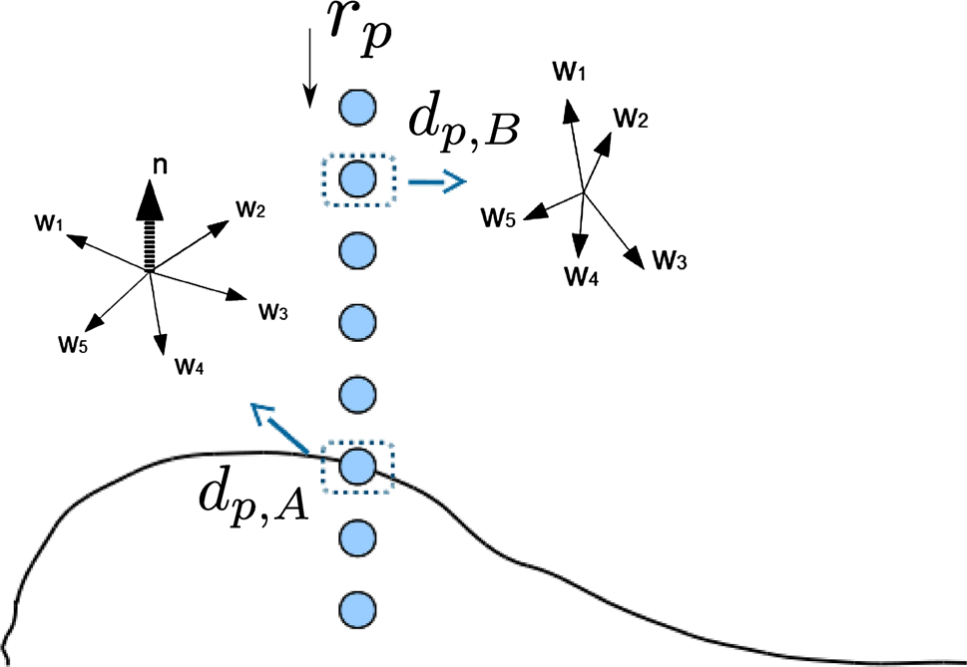
Maximum likelihood reconstruction in standard White Light Helmholtz Stereopsis: a conceptual illustration

The constraint is instrumental in the process of geometric reconstruction whereby for each surface point the most plausible depth is selected from a set of hypotheses. The principle of depth selection is illustrated in Fig. 3. Depth hypotheses $d_p$ are sampled along the projection ray $r_{p}$ to pixel *p* of the virtual camera (an orthographic one is shown for simplicity). For each $d_p$ constraints $\mathbf {w}\cdot \mathbf {n} = 0$ as in (3) are acquired in different reciprocal camera-light source configurations. As originally described in Zickler et al. (2002), in standard HS at least three constraints in the form $\mathbf {w}\cdot \mathbf {n} = 0$ are required in order to solve $W\mathbf {n} =0$ where *W* is the constraint matrix with $\mathbf {w}$ as rows. Singular value decomposition of *W*, i.e. $SVD(W) = U \Sigma V^{\top }$, gives a normal estimate $\mathbf {n}$ (the last column of *V*) and the confidence value for the estimate: $\frac{\sigma _2}{\sigma _3}$ where $\sigma _2$ and $\sigma _3$ are the second and third diagonal values of $\Sigma$ respectively. A high $\frac{\sigma _2}{\sigma _3}$ means that all constraints $\mathbf {w}$ are confined to two dimensions (i.e. co-planar), as at hypothesis $d_{p,A}$ in Fig. 3, with $\sigma _3$ tending to zero. The normal to the constraint plane in this case is well-defined. A low $\frac{\sigma _2}{\sigma _3}$, as at hypothesis $d_{p,B}$ in Fig. 3, would indicate a lack of constraint coplanarity due to the arbitrary $\mathbf {w}$ orientations. In reconstruction by HS, the depth hypothesis $d_p$ of the highest confidence value with its corresponding normal $\mathbf {n}$ is assigned to the surface location projecting to pixel *p*. In the standard formulation of HS the depth assignment is performed in a maximum likelihood (ML) manner i.e. optimising the depth at each surface location independently.

## Colour Helmholtz Stereopsis

We propose a novel approach that generalises Helmholtz Stereopsis (HS) to dynamic scenes—Colour HS (CL HS). In this section, the complete pipeline for CL HS featuring tailored calibration procedures and data processing algorithms is presented and formalised building on the notation presented in Sect. 3.

**Fig. 4:**
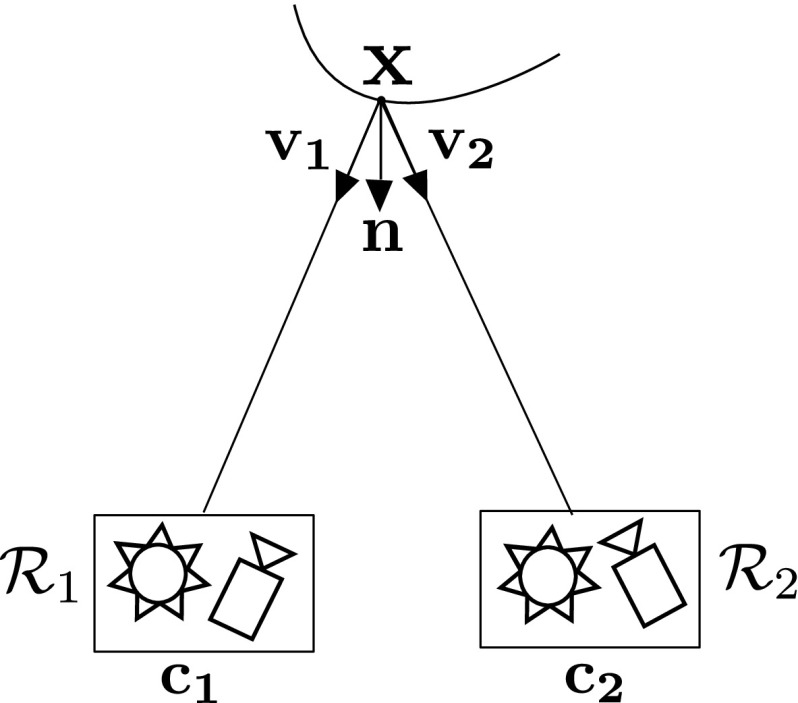
Reciprocal intensity sampling in Colour Helmholtz Stereopsis—physical camera-light source collocation

**Fig. 5:**
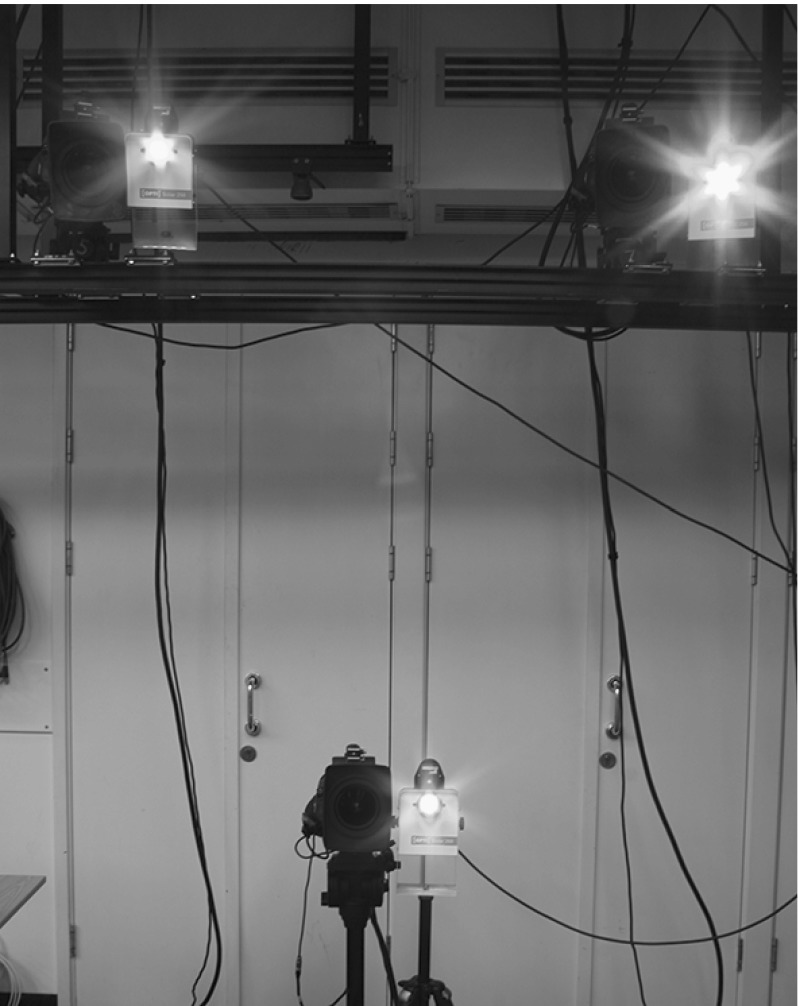
Experimental set-up of Colour Helmholtz Stereopsis

We expand the theory of WL HS to formalise CL HS. In CL HS, the $(\mathcal {C},\mathcal {S})$ pairs are realised by physical collocation (Fig. 4). The light sources such as $\mathcal {S}_{1}$ and $\mathcal {S}_{2}$ in Fig. 4 are characterised by different frequency spectra (Fig. 5). For consistent frequency-independent response, chromaticity of the reconstructed surface must be factored into the intensity equation. Unlike WL HS, in CL HS the illumination frequency $\omega$ cannot be omitted from the expression for the BRDF at $\mathbf {x}$: $f_{r}(\mathbf {v_2},\mathbf {v_1}, \omega )$. We propose to decompose BRDF $f_r$ into its directional component $f_{d}(\mathbf {v_2},\mathbf {v_1})$, dependent only on $\mathbf {v_{1}}$ and $\mathbf {v_{2}}$ (i.e. the viewing and illumination incidence vectors respectively) and the component related to the surface point chromaticity $p(\omega )$. We define the local chromatic constant $p_{1,2}$ as the reflectance coefficient due to the inherent colour of a point when seen by camera $\mathcal {C}_1$ and lit by light source $\mathcal {S}_2$. The camera is of importance due to possible differences in spectral sensor characteristics. For the coefficient to be 0, the illumination spectrum must exactly match the point’s chromatic absorption spectrum. This is unlikely to happen exactly, although the signal quality on non-dominant channels will degrade for points of purer (R, G or B) colours. Incorporating chromaticity $p_{1,2}$, we can re-write intensity equation in (1) for CL HS as:

4 $$\begin{aligned} i_{1} = \rho _{2}(\mathbf {v_{2}})\sigma _{1}(\mathbf {v_{1}})p_{1,2} f_{d}(\mathbf {v_2},\mathbf {v_1})\frac{ \mathbf {v_2} \cdot \mathbf {n} }{\Vert \mathbf {c_{2}} - \mathbf {x}\Vert ^2} \end{aligned}$$

For CL HS the normal constraint from (3) becomes:

5 $$\begin{aligned} \left( \frac{\mu _{1}(\mathbf {v_1}) i_{1} }{p_{1,2}\Vert \mathbf {c_{1}} - \mathbf {x}\Vert ^2} \mathbf {v_1} - \frac{\mu _{2}(\mathbf {v_2}) i_{2} }{p_{2,1}\Vert \mathbf {c_{2}} - \mathbf {x}\Vert ^2} \mathbf {v_2}\right) \cdot \mathbf {n}= 0 \end{aligned}$$

Note that no assumptions are made about the directional component of the BRDF $f_d$ - it can be arbitrary and unconstrainedly spatially-varying because due to the sampling configuration in HS consistency of the directional component within each reciprocal pair is guaranteed: $f_{d}(\mathbf{{v_2}},\mathbf{{v_1}}) = f_{d}(\mathbf {v_1},\mathbf {v_2})$. The constraint in (5) is formulated by making use of this $f_d$ equality, the knowledge of which stems from reciprocity, a fundamental property of the BRDF, and not from any surface homogeneity assumption.

**Fig. 6:**
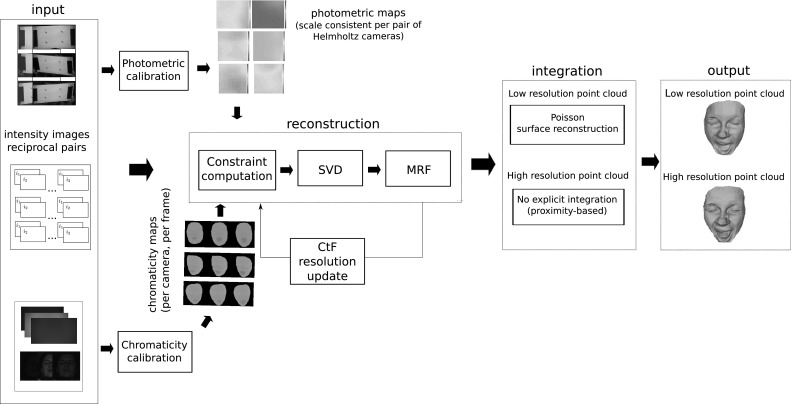
Overview of the pipeline for Colour Helmholtz Stereopsis (CL HS). The core part in the middle computes the point cloud by MRF optimisation based on the data term from SVD decomposition of CL HS constraints and a tailored prior. Depending on its resolution, the generated point cloud is integrated into a mesh either by Poisson surface reconstruction (low resolution point cloud) or, without explicit integration, by direct meshing of vertices by proximity, based on the known geometric relationships between them in the reconstruction volume (high resolution point cloud). The top and bottom branches of the pipeline are respectively the photometric and chromaticity calibration procedures essential for consistency in CL HS constraint formulation

Traditional HS set-ups feature just one camera-light source pair where either the equipment moves relative to the scene or the static scene is moved relative to the set-up for reciprocal pair acquisition. CL HS we propose is a static configuration consisting of three pairs of collocated cameras and light sources. The cameras are each equipped with an RGB sensor while the light sources all have different RGB characteristics. Each collocated pair of an RGB camera and a single-frequency-spectrum light source can be viewed as a *multi-spectral* Helmholtz camera (or, alternatively, as three single-frequency Helmholtz cameras of which only two are used in the set-up, as the camera will never receive the same frequency it transmits). The three light sources in the set-up must have the minimum frequency overlap to ensure signal separation. Signal separation allows simultaneous acquisition of the three required reciprocal pairs for normal estimation and enables generalisation to dynamic scenes.

An overview of the reconstruction pipeline for CL HS is given in Fig. 6. Bayesian HS from Roubtsova and Guillemaut (2014a) is the reconstruction core where depth labels are assigned in a global optimisation based on the residual of SVD decomposition of sampled HS constraints and a tailored prior. As well as the experimental set-up, Sect. 7 discusses Bayesian HS in more detail as a means of enabling high reconstruction accuracy under the restriction of three reciprocal pairs per frame. In contrast to WL HS, in CL HS spatially distributed and camera dependent photometric and chromaticity calibration parameters are essential to compute HS constraints. Photometric calibration is particularly important for CL HS due to the physically different cameras and multi-spectral light sources of the approach. Note that Helmholtz cameras in CL HS are essentially characterised as a sensor of one light frequency spectrum and a transmitter of another in different reciprocal pairs. Section 5 details how we generalise the algorithm from Jankó et al. (2004) for photometric calibration in CL HS and provides insights into its application in practice. Section 6 introduces the spatio-temporal procedure we devised for chromaticity calibration applicable to both static and dynamic scenes. The procedure eliminates intensity inconsistencies within the same reciprocal pair by a priori estimating surface chromaticity observed by each camera for the spectrum of each light source and, if necessary, propagating the parameters to any unseen frame.

**Fig. 7:**
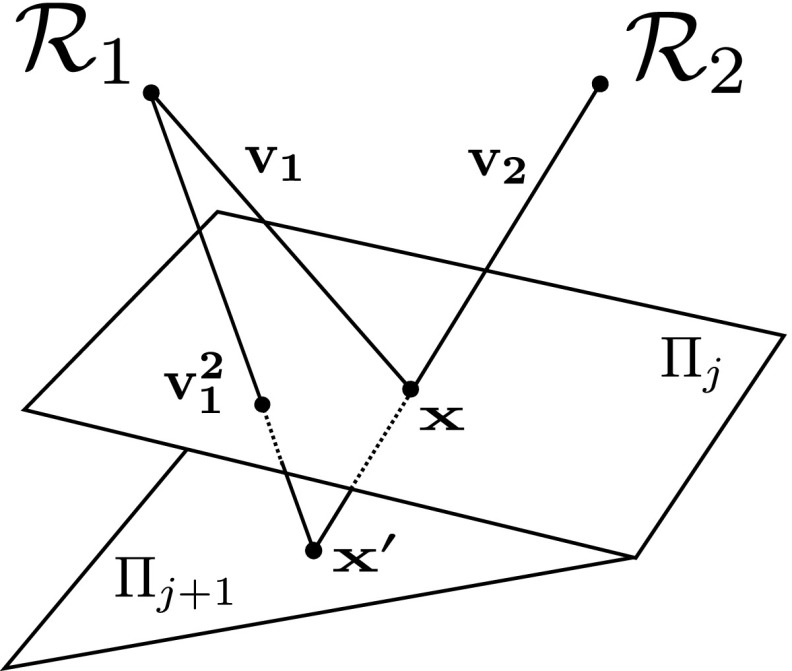
Geometry of the calibration procedure

## Helmholtz Camera Photometric Calibration 

**Fig. 8:**
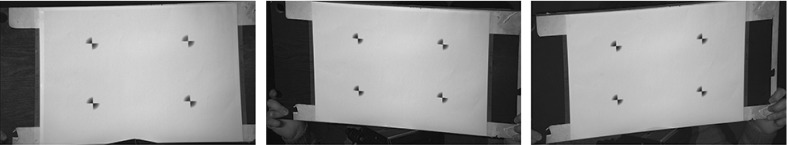
Calibration boards as viewed by $\mathcal {C}_{1}$, $\mathcal {C}_{2}$ and $\mathcal {C}_{3}$

Jankó et al. (2004) photometrically calibrate each Helmholtz camera $\mathcal {R}_{1}$ using another Helmholtz camera $\mathcal {R}_{2}$ by linking HS constraints, obtained by gradual displacement of a calibration plane, via the ray of incident illumination $\mathbf {v_{2}}$. In the original paper, the calibration was performed in a highly controlled environment with the plane translated in fixed vertical increments with a single camera and a light source suspended overhead and manually centred over the turntable with the plane (Fig. 2). For photometric calibration in the CL HS pipeline we went for a more freehand approach featuring a hand-held calibration board which was randomly moved within the reconstruction volume (Fig. 7). The calibration board bore four black markers (Fig. 8) for 3D plane localisation purposes. Specifically, detection, either manual or automatic, of at least three of those markers in the calibration images allows one to determine both the position and orientation of the calibration plane in 3D which defines the plane’s surface points $\mathbf {x}$ and normal $\mathbf {n}$ in the calibration equations in this section. Our entire configuration consisting of three multi-spectral Helmholtz cameras is calibrated simultaneously.

As in Jankó et al. (2004) for every position *j* of the calibration plane $\varPi _{j}$ we establish a ratio of parameters $\mu _{1}$ and $\mu _{2}$ corresponding to the Helmholtz cameras $\mathcal {R}_{1}$ and $\mathcal {R}_{2}$ sampled at a surface point $\mathbf {x}$ where rays $\mathbf {v_{1}}$ and $\mathbf {v_{2}}$ intersect (Fig. 7). However, in CL HS, the resultant ratio $\kappa$ is not the same as in WL HS as it is derived from (5) rather than (3) incorporating chromaticity:

6 $$\begin{aligned} \kappa (\mathbf {v_1}, \mathbf {v_2}\mid \varPi _j)=\frac{p_{2,1}}{p_{1,2}}\frac{\mu _{1}(\mathbf {v_{1}})}{\mu _{2}(\mathbf {v_{2}})}= \frac{\mathbf {n}^{\top }\mathbf {v_{2}}}{\mathbf {n}^{\top }\mathbf {v_{1}}}\frac{{\Vert \mathbf {c_{1}} - \mathbf {x}\Vert ^2}}{{\Vert \mathbf {c_{2}} - \mathbf {x}\Vert ^2}}\frac{i_{2}}{i_{1}} \end{aligned}$$

Effectively, in CL HS, we need to introduce the notion of *relative* photometric parameter distributions $\mu '_{1}=\frac{\mu _1}{p_{1,2}}$ and $\mu '_{2}=\frac{\mu _2}{p_{2,1}}$ capturing the radiometric properties of the acquisition equipment as sampled on the calibration object of a given *reference* chromaticity: $p_{1,2} = p^{ref}_{1,2}$ and $p_{2,1}=p^{ref}_{2,1}$. Thus $\kappa (\mathbf {v_1}, \mathbf {v_2}\mid \varPi _j)$ is a ratio of relative photometric distributions $\frac{\mu '_{1}}{\mu '_{2}}$. For simplicity, the chosen calibration object is of spatially uniform chromaticity (except the masked out black markers) i.e. $p^{ref}_{2,1}$ and $p^{ref}_{1,2}$ are constant for all surface points.

Subsequently, point $\mathbf {x}$ on plane $\varPi _{j}$ is transferred onto the plane in the new position $\varPi _{j+1}$ by finding the intersection $\mathbf {x}'$ of ray $\mathbf {v_{2}}$ with $\varPi _{j+1}$. Hence for plane $\varPi _{j+1}$ the ratio $\kappa ({\mathbf {v^{2}_{1}}, \mathbf {v_{2}}|\varPi _{j+1}}) =\frac{p_{2,1}\mu _{1}(\mathbf {v^{2}_{1}})}{p_{1,2}\mu _{2}(\mathbf {v_{2}})}$ is established sharing the denominator with the corresponding relationship of plane $\varPi _{j}$. The shared denominator, together with chromaticity constancy, allows to obtain a photometric parameter relationship $r_{1}(\mathbf {v_1}, \mathbf {v^{2}_{1}})$ between two pixel locations $(u_{1}, v_{1})$ and $(u^2_{1}, v^2_{1})$ corresponding to rays $\mathbf {v_1}$ and $\mathbf {v^{2}_{1}}$ in the spatial photometric distribution of $\mathcal {R}_{1}$:

7 $$\begin{aligned} r_{1}(\mathbf {v_1}, \mathbf {v^{2}_{1}}) =\frac{\kappa (\mathbf {v_1}, \mathbf {v_2}\mid \varPi _j)}{\kappa ({\mathbf {v^{2}_{1}}, \mathbf {v_{2}}|\varPi _{j+1}})} = \frac{p_{2,1}\mu _{1}(\mathbf {v_1})}{p_{2,1}\mu _{1}(\mathbf {v^{2}_{1}})} =\frac{\mu _{1}(\mathbf {v_1})}{\mu _{1}(\mathbf {v^{2}_{1}})} \end{aligned}$$

**Fig. 9:**
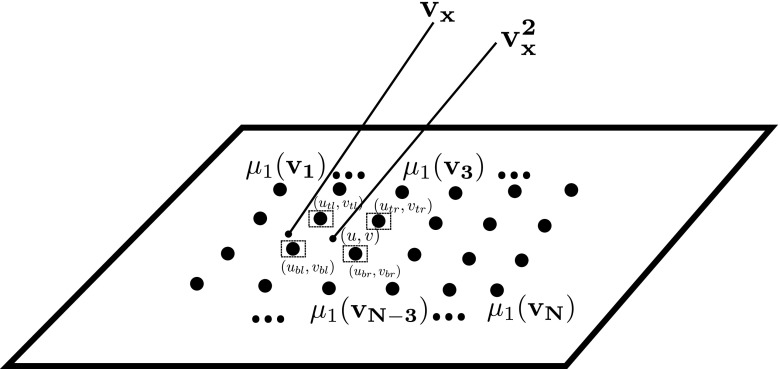
Estimation of the continuous spatially-varying photometric parameter distribution based on values $\mathbf {m_1}=[\mu _1( \mathbf { v_{1} } ),\mu _1( \mathbf { v_{2} } ), \ldots , \mu _1( \mathbf {v_{N}} )]$ of a regular grid of control points defined in the image domain. For example, the sample point at (*u*, *v*) corresponding to the sampling vector $\mathbf {v^{2}_x}$ will be expressed based on the $\mu _1$ distribution values at the four corners (*top-left*, *top-right*, *bottom-left* and *bottom-right* at respectively $(u_{tl},v_{tl})$, $(u_{tr},v_{tr})$, $(u_{bl},v_{bl})$ and $(u_{br},v_{br})$ ) of its control point grid square weighted by the bilinear interpolation coefficients. Vectors $\mathbf {v_x}$ and $\mathbf {v^{2}_x}$ define two linked samples in the photometric parameter distribution

Let us consider a uniformly sampled grid of control points $\mathbf {m_1}=[\mu _1( \mathbf { v_{1} } ),\mu _1( \mathbf { v_{2} } ), ... , \mu _1( \mathbf {v_{N}} )]$ sampled in the spatial photometric parameter distribution $\mu _1$ (see Fig. 9). Equation (7) provides constraints on the set of control points of the photometric map via bilinear interpolation in a linear system of equations. We chose to use a simpler regularisation kernel than Jankó et al. (2004) who perform bicubic interpolation between control points. Specifically, the natural logarithm of (7) is taken resulting in a single constraint on the control points of the form:

8 $$\begin{aligned} \lambda _{1} - \lambda _{2} = (\mathbf {a_i} -\mathbf {b_i})\varvec{\lambda } = \delta _i \end{aligned}$$

where $\lambda _{1} = \ln (\mu _{1}(\mathbf {v_x}))$ and $\lambda _{2} = \ln (\mu _{1}(\mathbf {v^2_x}))$ are the sample point values (see Fig. 9), $\varvec{\lambda } =[ \ln ( \mu _1( \mathbf { v_{1} } ) ) ,\ln ({ \mu _1( \mathbf { v_{2} } ) } ), ... ,\ln ({ \mu _1( \mathbf {v_{N} }) })]^\top$ is the vector of variables (control points), $\mathbf {a_i}$ and $\mathbf {b_i}$ are the bilinear interpolation coefficients from control points to sample points and lastly $\delta _i = \ln (r_{1}(\mathbf {v_x}, \mathbf {v^{2}_{x}}))$. Per sample point only four coefficients in interpolation vector $\mathbf {a_i}$ (or $\mathbf {b_i}$), specifically the ones corresponding to the four closest control points (Fig. 9), will be non-zero. Each point in the distribution is localised in the image domain by its (*u*, *v*) pixel coordinates - hence the four grid control points and the sample point coordinates are, respectively, $(u_{tl}, v_{tl})$, $(u_{tr}, v_{tr})$, $(u_{bl}, v_{bl})$, $(u_{br}, v_{br})$ and (*u*, *v*) where due to the grid sampling symmetry $u_{tl} = u_{bl}=u_{1}; u_{tr} = u_{br}=u_{2}$ and $v_{tl} = v_{tr}=v_{1}; v_{bl} = v_{br}=v_{2}$. With these definitions in mind, the corresponding four non-zero bilinear interpolation coefficients per sample point are defined as:

$${a_{tl}=\left( \frac{v_{2} - v}{v_{2} - v_{1}}\right) \left( \frac{u_{2} - u}{u_{2} - u_{1}}\right) }$$,

$${a_{tr}=\left( \frac{v_{2} - v}{v_{2} - v_{1}}\right) \left( \frac{u - u_{1}}{u_{2} - u_{1}}\right) }$$,

$${a_{bl}=\left( \frac{v - v_{1}}{v_{2} - v_{1}}\right) \left( \frac{u_{2} - u}{u_{2} - u_{1}}\right) }$$,

$${a_{br}=\left( \frac{v - v_{1} }{v_{2} - v_{1}}\right) \left( \frac{u - u_{1}}{u_{2} - u_{1}}\right) }$$.

From a set of constraints as in (8) the resultant linear system is:

9 $$\begin{aligned} (A - B)^\top (A - B)\varvec{\lambda } = (A - B)^\top \varDelta \end{aligned}$$

where $A =[\mathbf {a_0}, \mathbf {a_1},...,\mathbf {a_M}]^\top$, $B =[\mathbf {b_0}, \mathbf {b_1},...,\mathbf {b_M}]^\top$ and $\varDelta =[\delta _0, \delta _1 ..., \delta _{M}]^\top$. Having thus estimated all constrained control points of the grid, a continuous photometric distribution can be obtained by bilinear interpolation between them. In order to maximise the spatial coverage of the calibration, interpolation does not require all four neighbouring control points to be defined i.e. interpolation in the vertical or horizontal direction only is also permitted.

It has perhaps not been made explicit in Jankó et al. (2004) that Helmholtz cameras involved in a single reciprocal pair must be calibrated as a couple and not individually. Also, in CL HS, any pair of photometric distributions obtained will be relative to the calibration object (reference) chromaticity coefficients $p^{ref}_{1,2}$ and $p^{ref}_{2,1}$ as expressed in the ratio of (6). Specifically, control point values $\mathbf {m_1} = [\mu _1(\mathbf {v_1}),\mu _1(\mathbf {v_2}), ... , \mu _1(\mathbf {v_N})]$ on the spatial photometric parameter distribution of Helmholtz camera $\mathcal {R}_{1}$ are first obtained by taking the exponent of $\varvec{\lambda }$ from (9) and are subsequently bilinearly interpolated to form a continuous photometric distribution $\mu _1$. Then the photometric distribution $\mu _{1,2}$ (i.e. distribution 2 derived from distribution 1) for $\mathcal {R}_2$ is expressed by transfer of $\mu _1$ via (6):

10 $$\begin{aligned} \frac{p^{ref}_{1,2}}{p^{ref}_{2,1}}\mu _{1,2}(\mathbf {v_2}) = \frac{1}{\kappa (\mathbf {v_1}, \mathbf {v_2}\mid \varPi _j)}\mu _{1}(\mathbf {v_1}) \end{aligned}$$

We can define the relative transferred photometric distribution $\mu ''_{1,2} =\frac{p^{ref}_{1,2}}{p^{ref}_{2,1}}\mu _{1,2}$ where the transfer is from $\mathcal {R}_{1}$ to $\mathcal {R}_{2}$ and the relative aspect refers to the dependence of $\mu ''_{1,2}$ on the reference chromaticity. Hence, the calibrated pair of photometric distributions $(\mu _{1},\mu ''_{1,2})$ as a whole retains the dependence on the reference chromaticity of the calibration object. The dependence is equivalent to calibrating ratios $\mu '_1 =\frac{\mu _1}{p_{1,2}}$ and $\mu '_2 =\frac{\mu _2}{p_{2,1}}$ from Eq. (5) with $p_{1,2}= p^{ref}_{1,2}$ and $p_{2,1}= p^{ref}_{2,1}$ to result in:

11 $$\begin{aligned} \left( \frac{\mu '_{1}(\mathbf {v_1}) i_{1} }{\Vert \mathbf {c_{1}} - \mathbf {x}\Vert ^2} \mathbf {v_1} - \frac{\mu '_{2}(\mathbf {v_2}) i_{2} }{\Vert \mathbf {c_{2}} - \mathbf {x}\Vert ^2} \mathbf {v_2}\right) \cdot \mathbf {n}= 0. \end{aligned}$$

The equivalence of $(\mu _{1},\mu ''_{1,2})$ to $(\mu '_{1},\mu '_{2})$ can be observed by multiplying the homogeneous constraint in (11) through by $p^{ref}_{1,2}$. The relative photometric calibration parameters $\mu '_1$ and $\mu '_2$ fully calibrate the set-up for any surface of reference chromaticity. In Sect. 6.1, it will be shown how the undesirable dependence on the reference is neutralised in the HS normal constraint by a corresponding relative chromaticity estimation procedure removing all surface chromaticity limitations.

**Fig. 10:**
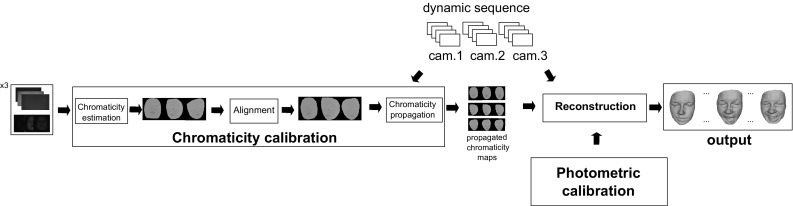
Spatio-temporal chromaticity calibration pipeline. Chromaticity in the reference frame is estimated per camera. The resultant chromaticity maps (i.e. spatial chromaticity distributions) are aligned with the reconstructed view. If a dynamic sequence is being reconstructed, the aligned chromaticity maps are temporally propagated throughout the dynamic sequence using dense point tracking by optical flow

Let us describe the procedure for distribution transfer from $\mu _1$ to $\mu ''_{1,2}$. Transferred control points $\mathbf {m''_{1,2}} = [\mu ''_{1,2}(\mathbf {v^*_1}),\mu ''_{1,2}(\mathbf {v^*_2}),...,\mu ''_{1,2}(\mathbf {v^*_N}) ]$ of the photometric calibration of $\mathcal {R}_2$ are defined by a set of sampling vectors $[\mathbf {v^*_1}, \mathbf {v^*_2} ... \mathbf {v^*_N}]$ (the asterisk is added to differentiate the vectors from those sampling the directly calibrated distribution). The expression relating a point from $\mathbf {m''_{1,2}}$ to the interpolated photometric map $\mu _1$ of $\mathcal {R}_{1}$ is:

12 $$\begin{aligned} \mu ''_{1,2}(\mathbf {v^*_1}) =\mu _{1,2}(\mathbf {v^*_1})\frac{p^{ref}_{1,2}}{p^{ref}_{2,1}} = \frac{1}{\kappa (\mathbf {v_1},\mathbf {v^*_1}\mid \varPi _j)}\mu _1(\mathbf {v_1}) \end{aligned}$$

where $\kappa (\mathbf {v_1}, \mathbf {v^*_1}\mid \varPi _j)$ is a single constraint for parameter transfer from $\mathcal {R}_{1}$ to $\mathcal {R}_2$. The constraint derived from sampling geometry and observed intensities of a point defined by the intersection of vectors $\mathbf {v_1}$ and $\mathbf {v^*_1}$ on a given calibration $\varPi _j$ is:

13 $$\begin{aligned} \kappa (\mathbf {v_1}, \mathbf {v^*_1}\mid \varPi _j) = \frac{\mathbf {n}^{\top }\mathbf {v^*_{1}}}{\mathbf {n}^{\top }\mathbf {v_{1}}}\frac{{\Vert \mathbf {c_{1}} - \mathbf {x}\Vert ^2}}{{\Vert \mathbf {c_{2}} - \mathbf {x}\Vert ^2}}\frac{i_{2}}{i_{1}} \end{aligned}$$

Note that $\mathbf {v_1}$ does not have to sample one of the control points of the spatial photometric parameter distribution $\mu _{1}$: any interpolated value of the distribution is a valid sample for the parameter transfer onto $\mathcal {R}_2$. Just as in the direct calibration process, to maximise support in the transfer linear system solved, multiple calibration planes $\varPi _j$ are used in the transfer and non-control points $\mu ''_{1,2}(\mathbf {v^*}) \notin \mathbf {m''_{1,2}}$ of the distribution $\mu ''_{1,2}$ also give rise to constraints (13). For every transfer sample, the computed $\kappa$ constrains up to four control points from $\mathbf {m''_{1,2}}$ that are related to the sample point through bilinear interpolation in the way defined in the N-dimensional constraint vector $\mathbf {k_i}$ (along with $\kappa$). The transfer linear system solved is:

14 $$\begin{aligned} K^\top K \varvec{m''_{1,2}} = K^\top \mathcal {M}_1 \end{aligned}$$

where $K = [\mathbf {k_1},\mathbf {k_2},...,\mathbf {k_M}]^\top$ and $\mathcal {M}_1 =[\mu ^1_{1}, \mu ^2_{1},..., \mu ^M_{1}]$ is a set of corresponding known *M* samples from the previously directly calibrated distribution $\mu _1$. Values $\mathbf {m''_{1,2}}$ obtained through the transfer procedure described are different from $\mathbf {m_{2}}$ that could have been computed by direct calibration of $\mathcal {R}_2$. The transfer procedure ensures mutual consistency of $\mathbf {m''_{1,2}}$ and the directly calibrated $\mathbf {m_{1}}$. Just as with the directly calibrated camera, the transferred control points are bilinearly interpolated between to produce a continuous spatial distribution.

In contrast to Jankó et al. (2004), we have found that for its accurate calibration a (multi-spectral) Helmholtz camera must be observed in at least two (multi-spectral) Helmholtz camera pairs as in Fig. 7. Jankó et al. (2004) mention ill-posedness of the calibration problem when a single Helmholtz camera pair is used due to constraints being sampled along the projection ray and the linked samples being along the same epipolar line. They however do not deem multiple Helmholtz camera pairs essential using a strong bicubic regulariser to address the ill-posedness. In our case, we make the problem better posed by making use of multiple pairs per multi-spectral Helmholtz camera which are readily available in the set-up. The better posedness allows us to work with a weaker bilinear regulariser thus avoiding potential artefacts due to over-regularisation. Calibration of a multi-spectral Helmholtz camera rather than a single-channel-sensitivity one, operates under the reasonable assumption of the shared spatial sensitivity distribution of the RGB sensors of a single physical camera $\mathcal {C}$ and simplifies the problem from six to just three directly calibrated unknown photometric distributions in the set-up (subsequently transferred for consistency onto their reciprocal pair partners as discussed).

**Fig. 11:**
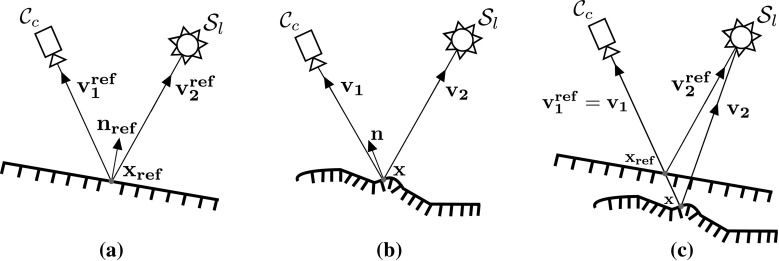
Reference and calibrated surface sampling for chromaticity estimation: **a** reference surface sampling; **b** calibrated surface sampling; **c** per-pixel relative chromaticity estimation

## Surface Chromaticity Calibration

In this section, we propose a spatio-temporal chromaticity calibration procedure applicable to dynamic as well as static scenes. The overview of the procedure’s pipeline is presented in Fig. 10. Its two main stages are the initial per camera chromaticity estimation in the reference frame (Sect. 6.1) and its subsequent temporal propagation (Sect. 6.2) to any new frame provided sufficient overlap with the reference. Both single-shot unseen (non-reference) static scenes as well as entire sequences in dynamic scene reconstruction can be served by the spatio-temporal chromaticity calibration approach. The method’s effectiveness in dynamic scene reconstruction is demonstrated in Sects. 8.2.2 and 8.2.3 of the evaluation.

### Initial Spatial Estimation

In this section, a procedure for per-pixel chromaticity estimation of the reconstructed surface is proposed. Since the three light sources in CL HS are red (R), green (G) and blue (B), the goal of the chromaticity calibration procedure is to compute the triplet $(p'_{c,R},p'_{c,G}, p'_{c,B})$ consisting of three chromaticity coefficients relative to the reference $(p^{ref}_{c,R},p^{ref}_{c,G},p^{ref}_{c,B})$. The triplet $(p'_{c,R},p'_{c,G}, p'_{c,B})$ describes the relative reflectance behaviour in response to red, green and blue illumination spectra for a visible surface point $\mathbf {x}$ viewed by camera $\mathcal {C}_c$ where $c=\{1,2,3\}$. It is assumed that the illumination spectra relate to the spectral sensor characteristics of the three cameras in the same way.

The calibration method is based on sampling the chromatic response of first a planar object with the chosen reference chromaticity $(p^{ref}_{c,R},p^{ref}_{c,G},p^{ref}_{c,B})$ and then that of the arbitrarily coloured object to be calibrated. Both the reference and the calibrated objects remain static during sampling to facilitate per-pixel estimation. For sampling of chromatic response, both objects are sequentially exposed to red, green and blue illumination from the same direction by changing colour filters of a single static light source. The colour filters used to sample chromatic response are subsequently intended for data acquisition at reconstruction. The sum of the RGB colour filter spectra defines white illumination in this context. Let us formalise chromaticity estimation per pixel of any $\mathcal {C}_c$ where $c=\{1,2,3\}$ since each pixel in the procedure is calibrated independently.

Consider the planar reference surface in Fig. 11a being sampled by camera $\mathcal {C}_c$ for any $c=\{1,2,3\}$ in the configuration of CL HS. A surface point on the reference object $\mathbf {x_{ref}}$ with the orientation $\mathbf {n_{ref}}$ projects onto the camera sensor pixel defined by $\mathbf {v^{ref}_1}$. The point is illuminated by a single light source $S_{l}$ in the fixed position whose inherent radiance distribution is sequentially coloured red ($\rho _{r}$), green ($\rho _{g}$) and blue ($\rho _{b}$) using filters for the purpose of chromatic response sampling of $\mathbf {x_{ref}}$. From (4) the image formation equations for the per-channel intensity responses $(i^{ref}_{c(r),r}, i^{ref}_{c(g),g}, i^{ref}_{c(b),b})$ at $\mathbf {x_{ref}}$ corresponding to the spectrum of illumination in each case are:

15 $$\begin{aligned} \begin{aligned} i^{ref}_{c(r),r} = \rho _{r}(\mathbf {v^{ref}_2})\sigma _{r}(\mathbf {v^{ref}_1})p^{ref}_{c,R} f_{d}(\mathbf {v^{ref}_2},\mathbf {v^{ref}_1})\frac{ \mathbf {v^{ref}_2} \cdot \mathbf {n_{ref}} }{\Vert \mathbf {c_{2}} - \mathbf {x_{ref}}\Vert ^2}\\ i^{ref}_{c(g),g} = \rho _{g}(\mathbf {v^{ref}_2})\sigma _{g}(\mathbf {v^{ref}_1})p^{ref}_{c,G} f_{d}(\mathbf {v^{ref}_2},\mathbf {v^{ref}_1})\frac{ \mathbf {v^{ref}_2} \cdot \mathbf {n_{ref}} }{\Vert \mathbf {c_{2}} - \mathbf {x_{ref}}\Vert ^2}\\ i^{ref}_{c(b),b} = \rho _{b}(\mathbf {v^{ref}_2})\sigma _{b}(\mathbf {v^{ref}_1})p^{ref}_{c,B} f_{d}(\mathbf {v^{ref}_2},\mathbf {v^{ref}_1})\frac{ \mathbf {v^{ref}_2} \cdot \mathbf {n_{ref}} }{\Vert \mathbf {c_{2}} - \mathbf {x_{ref}}\Vert ^2} \end{aligned} \end{aligned}$$

Note that triplet $(p^{ref}_{c,R},p^{ref}_{c,G}, p^{ref}_{c,B})$ is the chromaticity of the reference surface. This reference chromaticity must be the same as the chromaticity of the calibration plane in the photometric calibration procedure from Sect. 5 in order to ensure the reference independence of the CL HS pipeline as a whole. Typically, the reference tends to be chosen in the white spectrum of colours to maximise the channel response, although theoretically it does not have to be. In addition, the chromaticity of the reference object must be uniform since otherwise there will be per-pixel variations in the reference chromaticity, which would be impossible to reconcile with the reference in the photometric calibration.

Now consider the calibrated surface point $\mathbf {x}$ with orientation $\mathbf {n}$ in Fig. 11b sampled with the same three coloured radiance distributions $\rho _{r}$, $\rho _{g}$ and $\rho _{b}$ in an identical configuration of camera $C_{c}$ and light source $S_{l}$. The point-to-sensor projection is defined by vector $\mathbf {v_1}$ and the direction of illumination by $\mathbf {v_2}$. Each such point $\mathbf {x}$ on the calibrated object is also characterised by a triplet of image formation equations $(i_{c(r),r},i_{c(g),g}, i_{c(b),b})$ defined by its chromaticity $(p_{c,R},p_{c,G},p_{c,B})$ in response to the same stimuli:

16 $$\begin{aligned} i_{c(r),r}= & {} \rho _{r}(\mathbf {v_2})\sigma _{r}(\mathbf {v_1})p_{c,R} f_{d}(\mathbf {v_2},\mathbf {v_1})\frac{ \mathbf {v_2} \cdot \mathbf {n} }{\Vert \mathbf {c_{2}} - \mathbf {x}\Vert ^2}\nonumber \\ i_{c(g),g}= & {} \rho _{g}(\mathbf {v_2})\sigma _{g}(\mathbf {v_1})p_{c,G} f_{d}(\mathbf {v_2},\mathbf {v_1})\frac{ \mathbf {v_2} \cdot \mathbf {n} }{\Vert \mathbf {c_{2}} - \mathbf {x}\Vert ^2}\nonumber \\ i_{c(b),b}= & {} \rho _{b}(\mathbf {v_2})\sigma _{b}(\mathbf {v_1})p_{c,B} f_{d}(\mathbf {v_2},\mathbf {v_1})\frac{ \mathbf {v_2} \cdot \mathbf {n} }{\Vert \mathbf {c_{2}} - \mathbf {x}\Vert ^2} \end{aligned}$$

Note that $(p_{c,R},p_{c,G}, p_{c,B})$ in (16) is an absolute chromaticity triplet independent of any reference.

The camera-to-light source geometry is identical within each triplet of expressions in Eqs. (15) and (16) because both the set-up and the scene are static during sampling of each surface. This means that the ratio of any two expressions from (15) or (16) depends only on the relative $\rho$,$\sigma$ products and the corresponding per channel chromaticities. For example, the red-to-green response ratio for the reference surface point $\mathbf {x_{ref}}$ is:

17 $$\begin{aligned} \frac{i^{ref}_{c(r),r}}{i^{ref}_{c(g),g}} = \frac{\rho _r(\mathbf {v^{ref}_2})\sigma _r(\mathbf {v^{ref}_1})}{\rho _g(\mathbf {v^{ref}_2})\sigma _g(\mathbf {v^{ref}_1})}\frac{p^{ref}_{c,R}}{p^{ref}_{c,G}} \end{aligned}$$

The equivalent ratio for the calibrated surface point $\mathbf {x}$ is:

18 $$\begin{aligned} \frac{i_{c(r),r}}{i_{c(g),g}} = \frac{\rho _r(\mathbf {v_2})\sigma _r(\mathbf {v_1})p_{c,R}}{\rho _g(\mathbf {v_2})\sigma _g(\mathbf {v_1})p_{c,G}} \end{aligned}$$

Chromaticity is estimated per-pixel in the image domain of $\mathcal {C}_c$. Hence the idea is to link the reference and the calibrated surface points projecting onto the same pixel as shown in Fig. 11c. In other words, in the sampling configuration employed the projection vectors are the same: $\mathbf {v^{ref}_{1}} = \mathbf {v_1}$, and Eq. (17) simplifies to:

19 $$\begin{aligned} \frac{i^{ref}_{c(r),r}}{i^{ref}_{c(g),g}} = \frac{\rho _r(\mathbf {v^{ref}_2})\sigma _r(\mathbf {v_1})}{\rho _g(\mathbf {v^{ref}_2})\sigma _g(\mathbf {v_1})}\frac{p^{ref}_{c,R}}{p^{ref}_{c,G}} \end{aligned}$$

meaning that the same point in the sensitivity distributions $\sigma _r$ and $\sigma _g$ applies to the corresponding reference and calibrated surface points, $\mathbf {x_{ref}}$ and $\mathbf {x}$ respectively. However, Fig. 11c also shows that the illumination vectors $\mathbf {v^{ref}_{2}}$ and $\mathbf {v_{2}}$ are clearly not the same because the sampled 3D points $\mathbf {x_{ref}}$ and $\mathbf {x}$ are not identical.

**Fig. 12:**
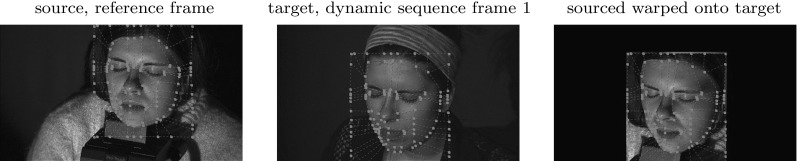
Alignment of the reference frame to the first dynamic sequence frame (camera 2). The superimposed feature grids were manually initialised in the source and target frames. Local (piecewise) homography with bicubic interpolation is used to warp the source frame onto the target frame

An important simplification can be made to Eqs. (18) and (19) given that the coloured radiance distributions $\rho _{r}$, $\rho _{g}$ and $\rho _{b}$ are realised by applying colour filters to a radiance distribution of a single light source $S_{l}$. Colour filters can be assumed spatially uniform which means that the radiance distributions $\rho _{r}$, $\rho _{g}$ and $\rho _{b}$ differ from each other by a constant scale factor. For example, for one pair of distributions one can write: $\rho _{r} = k\rho _{g}$ where *k* is a constant meaning that the same relationship holds for any two spatially corresponding samples of the distributions. With this in mind, the ratio in (19) can be re-written as:

20 $$\begin{aligned} \frac{i^{ref}_{c(r),r}}{i^{ref}_{c(g),g}} = \frac{k\rho _g(\mathbf {v^{ref}_2})\sigma _r(\mathbf {v_1})}{\rho _g(\mathbf {v^{ref}_2})\sigma _g(\mathbf {v_1})}\frac{p^{ref}_{c,R}}{p^{ref}_{c,G}} = k\frac{\sigma _r(\mathbf {v_1})}{\sigma _g(\mathbf {v_1})}\frac{p^{ref}_{c,R}}{p^{ref}_{c,G}}. \end{aligned}$$

Equivalently, (18) becomes:

21 $$\begin{aligned} \frac{i_{c(r),r}}{i_{c(g),g}} = \frac{k\rho _g(\mathbf {v_2})\sigma _r(\mathbf {v_1})p_{c,R}}{\rho _g(\mathbf {v_2})\sigma _g(\mathbf {v_1})p_{c,G}} = k\frac{\sigma _r(\mathbf {v_1})}{\sigma _g(\mathbf {v_1})}\frac{p_{c,R}}{p_{c,G}}. \end{aligned}$$

As a result of the simplification, Eqs. (20) and (21) can be combined by substitution for $k\frac{\sigma _r(\mathbf {v_1})}{\sigma _g(\mathbf {v_1})}$ and one obtains an expression for the ratio of two relative chromaticity components $p'_{c,R}$ and $p'_{c,G}$, defined against the reference chromaticity components $p^{ref}_{c,R}$ and $p^{ref}_{c,G}$, as a function of directly measurable intensities:

22 $$\begin{aligned} \frac{p'_{c,R}}{p'_{c,G}} = \frac{ \frac{ p_{c,R} }{ p^{ref}_{c,R} } }{ \frac{ p_{c,G} }{ p^{ref}_{c,G} } } = \frac{i_{c(r),r}}{i_{c(g),g}}\frac{i^{ref}_{c(g),g}}{i^{ref}_{c(r),r}} \end{aligned}$$

Three such ratios, e.g. $\frac{ p'_{c,R} }{ p'_{c,G} }$, $\frac{ p'_{c,R} }{ p'_{c,B} }$ and $\frac{ p'_{c,G} }{ p'_{c,B} }$,:

23 $$\begin{aligned} \begin{aligned} \frac{p'_{c,R}}{p'_{c,G}} = \frac{i_{c(r),r}}{i_{c(g),g}}\frac{i^{ref}_{c(g),g}}{i^{ref}_{c(r),r}} = c_{1} \\ \frac{p'_{c,R}}{p'_{c,B}} = \frac{i_{c(r),r}}{i_{c(b),b}}\frac{i^{ref}_{c(b),b}}{i^{ref}_{c(r),r}} = c_{2} \\ \frac{p'_{c,G}}{p'_{c,B}} = \frac{i_{c(g),g}}{i_{c(b),b}}\frac{i^{ref}_{c(b),b}}{i^{ref}_{c(g),g}} = c_{3} \end{aligned} \end{aligned}$$

result in three homogeneous constraints constituting a homogeneous linear system of equations:

24 $$\begin{aligned} \left( \begin{array}{ccc} 1 &{} -c_1 &{} 0 \\ 1 &{} 0 &{} -c_{2} \\ 0 &{} 1 &{} -c_3 \end{array} \right) \left( \begin{array}{c} p'_{c,R} \\ p'_{c,G} \\ p'_{c,B} \\ \end{array} \right) = 0. \end{aligned}$$

The system is solved by SVD decomposition of the constraint matrix with as the solution a normalised vector i.e. the chromaticity coefficient triplet $[p'_{c,R}, p'_{c,G}, p'_{c,B}]^\top$.

Substitution of the relative chromaticity coefficients $p'_{1,2} = \frac{p_{1,2}}{p^{ref}_{1,2}}$ and $p'_{2,1} = \frac{p_{2,1}}{p^{ref}_{2,1}}$, where $p_{c,l}$ is defined by the camera $c=\{1,2,3\}$ and light source $l=\{R,G,B\}$, together with the relative photometric parameter distributions $\mu '_1 = \frac{\mu _1}{p^{ref}_{1,2}}$ and $\mu '_2=\frac{\mu _2}{p^{ref}_{2,1}}$ from Sect. 5 into Eq. (5) instead of the absolute values:

25 $$\begin{aligned} \left( \frac{p^{ref}_{1,2}\mu _{1}(\mathbf {v_1}) i_{1} }{p_{1,2}p^{ref}_{1,2}\Vert \mathbf {c_{1}} - \mathbf {x}\Vert ^2} \mathbf {v_1} - \frac{p^{ref}_{2,1}\mu _{2}(\mathbf {v_2}) i_{2} }{p_{2,1}p^{ref}_{2,1}\Vert \mathbf {c_{2}} - \mathbf {x}\Vert ^2} \mathbf {v_2}\right) \cdot \mathbf {n}= 0 \end{aligned}$$

results in the cancellation of the reference chromaticity from the normal constraint equation. Hence a constraint where both photometric and chromaticity calibration are relative to the same reference chromaticity:

26 $$\begin{aligned} \left( \frac{\mu '_{1}(\mathbf {v_1}) i_{1} }{p'_{1,2}\Vert \mathbf {c_{1}} - \mathbf {x}\Vert ^2} \mathbf {v_1} - \frac{\mu '_{2}(\mathbf {v_2}) i_{2} }{p'_{2,1}\Vert \mathbf {c_{2}} - \mathbf {x}\Vert ^2} \mathbf {v_2}\right) \cdot \mathbf {n}= 0 \end{aligned}$$

is equivalent to the fundamental normal constraint of HS in Eq. 5 formulated in terms of the absolute values not directly accessible. To take advantage of this equivalence the same reference chromaticity must be used in both the photometric calibration of Sect. 5 and the chromaticity estimation described in this section.

Chromaticity describes only the relative inter-channel relationship, not the absolute intensities, which means that multiple colours map onto the same chromaticity (e.g. all greyscale values are the same in terms of chromaticity). The estimation procedure describes each set of colours with the same inter-channel relationship by a single colour from the set, specifically the one corresponding to the normalised vector $[p'_{c,R}, p'_{c,G}, p'_{c,B}]^\top$. For example, due to this intensity ambiguity, all greyscale colours map onto the RGB triplet $[\frac{1}{\sqrt{3}}, \frac{1}{\sqrt{3}}, \frac{1}{\sqrt{3}}]$, which is the normalised vector expressing inter-channel equality. It should be stressed that disambiguation of colours with the same chromaticity is irrelevant for reconstruction by CL HS. The normal constraint of CL HS in Eq. (26) is homogeneous meaning that any consistent scaling of chromaticity coefficients cancels out.

### Temporal Propagation

The chromaticity calibration procedure described in the previous section is static with non-instantaneous acquisition, mapping chromaticities to pixel locations in one particular frame. For reconstruction of dynamic sequences with spatially varying chromaticity per-pixel colour information is needed for every frame. We propose to infer chromaticity in each frame of the dynamic sequence from the original single-frame calibration result using a chromaticity propagation procedure.

#### Optical Flow Based Procedure

As pre-processing, the chromaticity map of the reference frame must be aligned with the first frame of the dynamic sequence in order to form the starting point for chromaticity propagation. In the simplest case, the calibration would immediately precede the dynamic capture and the calibration shot will be roughly the same as the first frame of the sequence. Unaided perfect alignment occurs only in the case of static reconstruction of the reference frame used for chromaticity estimation. Any minor misalignment can be corrected using optical flow techniques. However, one would not wish to be limited to reconstruction of just the tailored dynamic sequence and hence re-use of chromaticity data for dynamic sequences featuring the same object but different initial positions is desirable. The problem of larger viewpoint variation with such untailored dynamic sequences is solved by warping the calibration shot (the source) to the initial frame of the dynamic sequence (the target) provided a sufficient degree of overlap between the two. The warping procedure involves initialisation of corresponding feature points in both the source and target and a transformation to align the features. The nature of the transform used depends on the surface being aligned. For (near-)planar surfaces, a global homography may be sufficient whereas surfaces with local curvatures require more sophisticated forms of warping. For alignment of non-planar surfaces, the user is required to define a coarse grid whose vertices correspond to scene features. Warping is then performed by local (piecewise) homography with bicubic interpolation. Non-planar alignment is illustrated in Fig. 12 that shows the source and target intensity images with the manually initialised feature grids superimposed and the resultant warping of the source onto the target. The approach has been found capable of coping with a visually substantial difference between the source and target. The alignment stage of the propagation process is the only part of the pipeline requiring manual interaction for feature matching. This requires minimal user effort and provides the ability to process multiple dynamic sequences with substantially varying initial poses from a single calibration result.

**Fig. 13:**
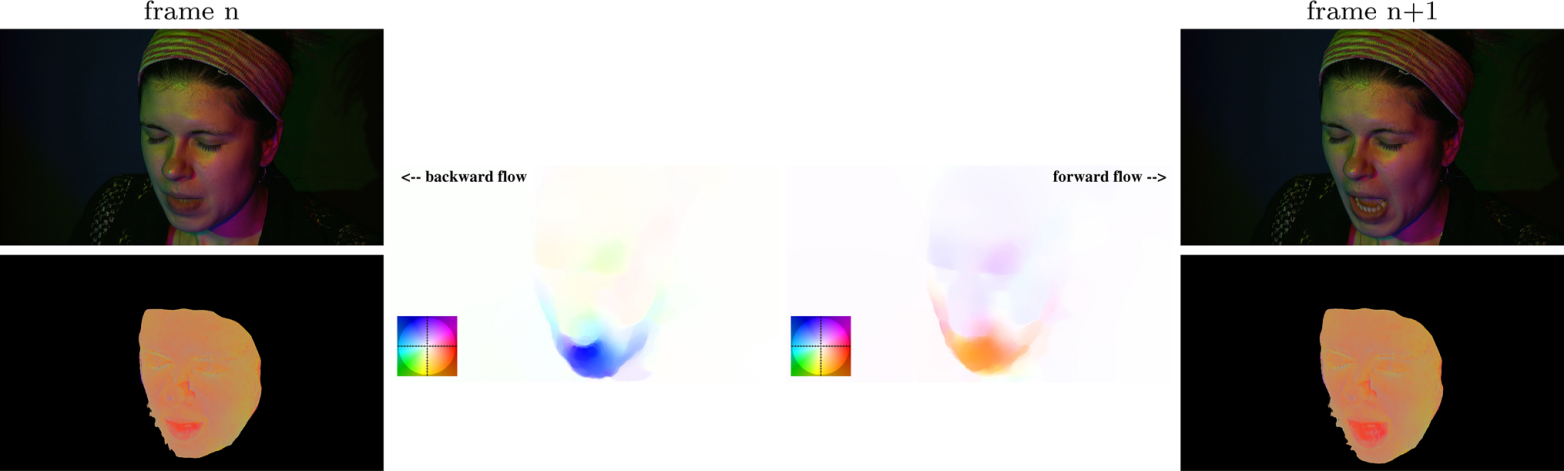
Chromaticity propagation between a pair of adjacent frames. The backward and forward flow maps are computed based on intensity images. The chromaticity of frame $n+1$ is derived from that of frame *n* using the backward flow map

As the first stage of propagation, dense optical flow tracking is performed on the intensity images of the dynamic sequence for each camera separately. We use the efficient GPU implementation of optical flow from Sundaram et al. (2010), which has the large displacement optical flow (LDOF) (Brox et al. 2009) in its core. LDOF is a variational technique with a continuous energy functional whose optimisation is embedded into a coarse-to-fine framework allowing one to estimate large displacements even for smaller scene components (Brox et al. 2009). The ability of the algorithm to cover a wide range of displacement amplitudes permits freedom in the choice of motion speed and frame rates of the tracked dynamic scenes.

Tracking produces per-pixel flow maps (Baker et al. 2011) that are used in our work to propagate the aligned chromaticity calibration throughout the dynamic sequence, effectively establishing the mapping of the calibrated chromaticities to each frame. To ensure completeness of mapping coverage, backward flows are utilised rather than forward flows, meaning that each pixel of the current frame is assigned the chromaticity triplet (if defined) of the quantised to the nearest pixel back-projection in the previous frame. The nearest neighbour approach is chosen over higher order interpolation in order to avoid colour blurring at region boundaries of the chromaticity map. To illustrate the process, Fig. 13 shows, for one particular camera, two adjacent intensity frames with a substantial relative motion, propagation of chromaticity between the two and the corresponding backward and forward flow maps. The process of propagation hence provides a spatially varying chromaticity estimate for each frame of the dynamic sequence.

#### Propagation Error

It is true that optical flow computation is not an easy problem for real dynamic scenes which will inevitably introduce errors into chromaticity propagation. The errors have not been found prohibitive for the performance of the proposed system because of several reasons. Firstly, optical flow is known to err mainly in the uniform intensity regions due to the inherent ambiguity. Since uniform intensity generally correlates well with uniform chromaticity, a frame-to-frame misprojection within a single constant intensity region may not be noticeable in the propagated chromaticity map as the chromaticity is also the same throughout the region. Secondly, the Bayesian reconstruction core with a tailored depth-normal consistency prior of the proposed system (see Sect. 7.2 for details) offers a significant degree of robustness to various signal perturbation such as chromaticity propagation errors and cross-talk (see Sect. 7.3).

There are several things that could potentially be implemented to reduce the optical flow error, especially the drift problem for longer dynamic sequences. The most obvious device would be re-initialisation of the chromaticity map at regular intervals in the sequence, particularly when the subject pose changes significantly. Further, non-sequential tracking methods can be used that exploit similarity of pose within groups of not necessarily consecutive frames in a sequence by constructing a minimum spanning tree. The technique has been been successfully utilised for registration of mesh sequences involving non-rigid surface deformation (Klaudiny et al. (2012); Budd et al. (2013)) but can be equally applicable in 2D tracking. Finally, the propagation of chromaticity to a given frame can be robustified by making use of the reconstructed geometry from the preceding frames. Although propagation cannot be heavily based on prior geometry projection due to the possibility of abrupt pose variation, the error relative to the chromaticity prediction by projection can certainly act as an extra term, in addition to the optical flow cost, in the energy optimisation of per-frame chromaticity estimate.

## Implementation 

In this section, we discuss the details of CL HS implementation at various stages of the pipeline from acquisition to reconstruction. Further, cross-talk in the multi-spectral acquisition system is discussed as a source of error with a corresponding estimate of signal corruption and practical tips to minimise its influence.

### Acquisition

Figure 5 shows our acquisition set-up consisting of three pairs of collocated RGB cameras $\mathcal {C}_{c}$ where $c=\{1,2,3\}$ and light sources $\mathcal {S}_l$ where $l =\{r,g,b\}$. Due to the use of RGB cameras, each collocated pair is a multi-spectral Helmholtz camera or a triplet of single-sensor-frequency Helmholtz cameras $\mathcal {R}_{c,l}$ defined by the light spectrum transmitter collocated with the RGB camera at position *c* and the camera sensor of frequency channel *l*. Only two Helmholtz cameras of each collocated pair are used at acquisition resulting in a set-up consisting of the total of six Helmholtz cameras: $( \mathcal {R}_{1,r}, \mathcal {R}_{1,g}, \mathcal {R}_{2,b}, \mathcal {R}_{2,g}, \mathcal {R}_{3,r}, \mathcal {R}_{3,b} )$. Sources $\mathcal {S}_l$ are given different frequency characteristics by using red, green and blue colour filters for maximal spectral separation. The filters were chosen to match RGB channel spectra of the cameras as much as possible and no ambient light is allowed. With the set-up we simultaneously acquire three reciprocal image pairs, each characterised by two Helmholtz cameras and two RGB signal channels.

### Reconstruction

Using these reciprocal pairs, constraints as in (5) are formulated. The constraints can be directly integrated into the original reconstruction pipeline proposed by Zickler et al. (2002) in the seed paper introducing HS. However, standard ML HS is known to be prone to noise due to the lack of regional support in depth assignment. In the proposed CL HS we are inherently limited to just three reciprocal pairs leaving room for reconstruction ambiguity. Additional intensity error may occur through channel cross-talk which we do not explicitly compensate for in this work. Consequently, standard ML HS is inadequate in this case. In this work, we adopt the Bayesian formulation of HS from our previous publications (Roubtsova and Guillemaut 2014a, 2015) where depth assignment is performed by minimising the joint sum of data ($E_{data}$) and prior ($E_{prior}$) costs over all surface points. To define a set of reconstruction variables, the surface is sampled spatially with a grid of pixels *p* of an orthographic virtual camera which determines the reconstruction view. The virtual camera pixel grid defines spatial neighbourhoods $\mathcal {N}(p)$ where continuity is enforced by a prior in the optimisation process. Depth hypotheses $d_p$ for each virtual camera pixel *p* are sampled along its orthographic projection ray through the scene. Bayesian HS seeks the optimum depth labelling configuration $f_{MAP}$ in the set *S*:

27 $$\begin{aligned} f_{MAP}^{*} = \underset{f\in S}{{\text {argmin}}}\sum \limits _{{p}} ( (1 - \alpha )E_{data}(p,d_{p})\; \nonumber \\ + \sum \limits _{p'\in \mathcal N(p)} \alpha E_{prior}(p,d_{p}, p',d_{p'}) ) \end{aligned}$$

where $\alpha$ is a weighting factor for the relative contributions of the two terms. The data term is computed via SVD decomposition of the matrix consisting of three CL HS constraints (5) instantaneously acquired with our set-up. Specifically, the term is defined as the exponential decay with factor $\mu = 0.2\ln (2)$ of the SVD residual quotient:

28 $$\begin{aligned} E_{data}(p,d_{p}) = e^{ -\mu \times \frac{\sigma _{2}(p,d_{p})}{\sigma _{3}(p,d_{p})} } \end{aligned}$$

The chosen prior is based on the idea from Roubtsova and Guillemaut (2014a) to enforce consistency between depth *d* and normal $\mathbf {n}$ estimates of neighbouring points (please, refer to the publication for more detail). The prior is uniquely tailored to HS as this method inherently associates a normal $\mathbf {n}$ with every depth hypothesis $d_{p}$ of virtual camera pixel *p* via SVD decomposition. The prior minimises the consistency error between the geometric local surface curvature and the photometric normal estimates:

29 $$\begin{aligned} E_{prior}(p,d_{p}, p',d_{p'}) = {err}(d_{p},n_{p},d_{p'}, n_{p'}) \end{aligned}$$

The depth-normal consistency prior has been shown in Roubtsova and Guillemaut (2014a) to result in the most accurate depth maps compared to one-sided (depth-based or normal-based) priors.

The optimisation process results in a point cloud of oriented vertices whose resolution can be controlled by embedding the reconstruction core into a coarse-to-fine framework with both spatial and depthwise subdivision at each iteration. By harnessing the joint advantage of the tailored depth-normal consistency prior and the coarse-to-fine approach one can generate such accurate high resolution point clouds that can be meshed directly without explicit integration as post-processing. The direct meshing is accomplished by knowing the proximity relationships between vertices in the reconstruction volume. If the resolution of the point cloud is insufficient for direct meshing, integration can be performed by Poisson surface reconstruction (Kazhdan et al. 2006). Note that the generated point cloud is only a 2.5D sampling of the object with its back-side being occluded. Poisson surface reconstruction requires a full 3D point cloud, otherwise it is unable to close the surface, which distorts the reconstructed view. Inspired by the approach in Vlasic et al. (2009), for integration with Poisson surface reconstruction, the 2.5D point cloud with the occluded back-end is extended to obtain a watertight full 3D cloud taking normal orientation cues from the contour of the object’s orthographic projection to the virtual camera. The point cloud extension to full 3D is implemented merely to make the integration problem solvable by Poisson surface reconstruction and is not representative of the true geometry of the occluded back-end. The need for such speculative point cloud completion as well as the danger that integration as a post-processing step may introduce artefacts (e.g. point cloud over-smoothing) are the reasons why this explicit surface integration should best be avoided if possible. The relative performance of Poisson surface reconstruction and the advocated no explicit integration approach is compared on static scenes in Sect. 8.1.1 to support the statements made on the applicability of each.

### Cross-Talk

Cross-talk occurs when a fraction of a signal intended for one channel is received on another channel. In the presented system based on wavelength-multiplexing cross-talk is the RGB illumination signals exciting the wrong camera sensors. The energy may be thus lost to other channel sensors or received from an unintended stimulus. Either way the received signal on each individual channel can be distorted by cross-talk.

A way to spatially estimate cross-talk is to measure the response of the three multi-spectral Helmholtz cameras in the reconstruction configuration to red, green and blue light reflected from a white surface. Assuming a perfectly white surface, the signal of a given colour in theory should excite a response only in the corresponding channel. The sum of responses on the other two channels is cross-talk. Measuring the response in the reconstruction configuration allows one to estimate a spatial distribution of cross-talk percentage from the total signal strength for a real-life scenario. Such a map does not allow to correct for cross-talk at reconstruction as the chromatic properties of the reconstructed surface will change the distribution. However, the distribution can serve as a good indication of the quality of the Helmholtz camera configuration.

**Fig. 14:**
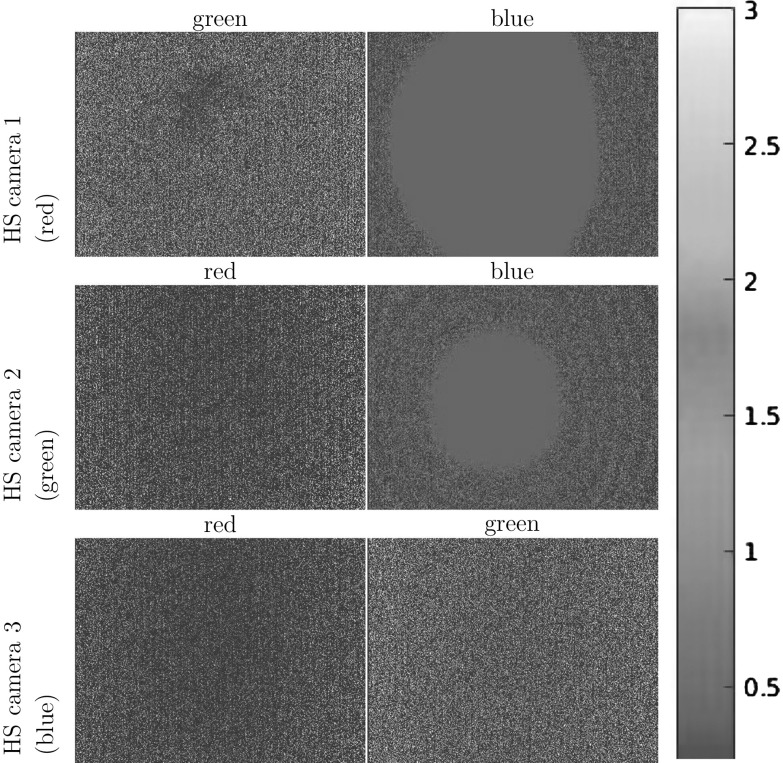
Spatial distribution of cross-talk as a percentage of the total signal per pixel in an example reconstruction configuration

Let us consider an example acquisition equipment configuration of three multi-spectral Helmholtz cameras (one less advantageous than the actual reconstruction configuration used) that helps provide practical suggestions for a better set-up in terms of cross-talk. For each camera the cross-talk percentage is measured under two signal wavelengths (the third wavelength is the one the Helmholtz camera emits itself and hence is irrelevant). Cross-talk is represented as a spatial distribution of the percentage leaked signal from the total signal strength. Fig. 14 shows the six distributions in the three camera reconstruction configuration with the corresponding statistical metrics given in Table 1. Generally speaking, the rms cross-talk observed in this configuration is about $1-2\,\%$ (which with an 8-bit camera sensor amounts to about 5 intensity levels with a single light source in the test configuration). As the general trend the measured cross-talk percentage tends to increase towards the outskirts of the frame as the total signal strength there tends to be substantially weaker with essentially a low signal-to-(cross-talk) noise ratio. The drastic cross-talk percentage maxima given in Table 1 are not reliable measurements as they occur at near-zero signal intensity levels.

The cross-talk percentage expresses the relative significance of signal leakage in perturbing reconstruction and is co-determined by the local signal strength as well as the overall camera-light source RGB spectra compatibility. To minimise the effect of cross-talk at reconstruction the signal-to-noise ratio must be kept high in the region of interest. In other words the scene should be optimally illuminated. From the distribution presented in Fig. 14 it is clear that the blue light in the example configuration is oriented the most advantageously with the scene well-lit in a clearly defined spotlight where the locally observed cross-talk percentage is under $0.5\,\%$. The orientations of the green and red light sources are inferior.

**Table 1:** Cross-talk statistics: rms and maximum cross-talk percentages of the spatial distributions presented in Fig. 14

HS camera 1 (red)		
Stimulus	rms cross-talk (%)	max. cross-talk (%)
Green	2.14	60
Blue	0.87	40
HS camera 2 (green)		
Stimulus	rms cross-talk (%)	max. cross-talk (%)
Red	1.54	53.85
Blue	0.88	40
HS camera 3 (blue)		
Stimulus	rms cross-talk (%)	max. cross-talk (%)
Red	1.34	53.33
Green	2.12	60

With a proper light source positioning resembling that of the blue light in the example configuration, cross-talk is unlikely to pose reconstruction challenges. In setting up the reconstruction configuration care must be taken to ensure that the scene receives the maximum amount of light from all the three light sources in order to minimise the cross-talk percentage (or equivalently to maximise the signal-to-noise ratio) in the reconstruction volume. Such a configuration can be easily achieved during scene framing by collocating the spatial centre of the reconstruction volume with the optical spotlight axes of all the projectors. Given such a configuration, the system will be able to cope well with any residual cross-talk (e.g. the rms value of $1-2\,\%$ in Table 1) by virtue of the optimal Bayesian HS reconstruction core with its effective depth-normal consistency prior.

## Evaluation

The methodology of CL HS is validated using real datasets with the evaluation comprising static and dynamic scenes versatile in the challenges they present.

In Sect. 8.1, the range of static objects was selected to demonstrate accuracy of the algorithm and its ability to cope with cases of different complexity. Shown in Fig. 18, the objects are: 1. a reference chromaticity plane (“Plane”) defined by the calibration board; 2. a plaster statue of a monster head (“Monster”) of not strictly uniform chromaticity; 3. a highly specular mug (“Mug”) of reference chromaticity and 4.  toy-dog (“Slinky”) which is highly heterogeneous in terms of material, chromaticity, reflectance etc. and shows geometric and radiometric complexity due to fine structure, transparency, specular reflectance and pure colours of its various materials.

Having shown correctness of geometric reconstruction for the more controlled static scenery sets, we subsequently validate our claim of suitability of CL HS for dynamic scene reconstruction. We are particularly interested in reconstruction of such scenes with complex reflectance properties as these are inherently challenging for conventional and photometric stereo methods. In the dynamic scene evaluation in Sect. 8.2 a range of temporal object deformations was reconstructed featuring objects posing distinctive geometric and photometric challenges. The objects are a highly specular laminated white sheet (“WLS”), a white glossy blouse (“Blouse”), a woollen jumper with structural detail of its approximately uniformly coloured knitwear (“Jumper”) and a face showing reflectance complexity with its spatially varying chromaticity and non-Lambertian directional component (“Face”). As will be explained in Sect. 8.2, the selection of objects covers the full spectrum of possible surface chromaticity variation from uniformly reference chromaticity to freely spatially varying.

**Fig. 15:**
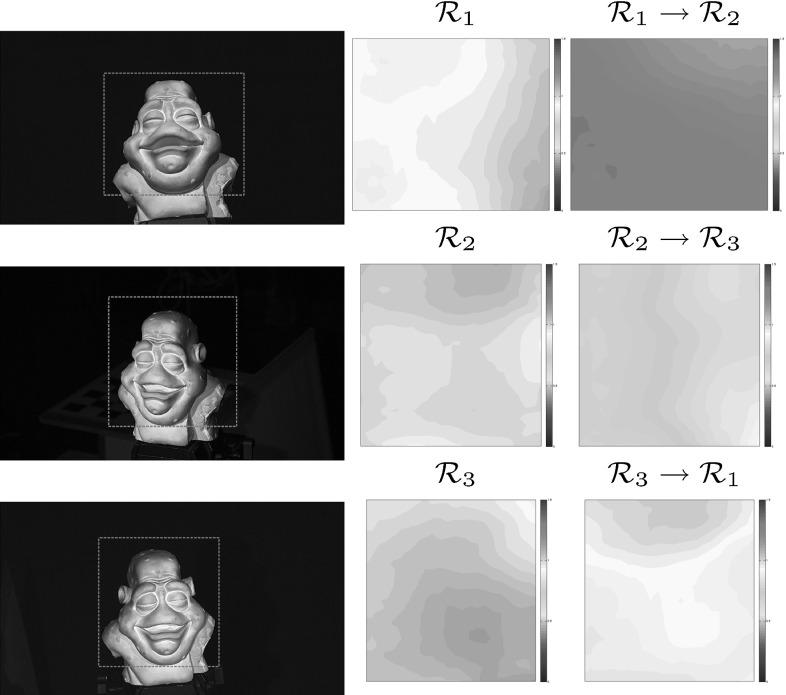
Pairwise (per reciprocal pair) consistent photometric parameter distributions in the region of interest for Monster. $(\mathcal {R}_{1}, \mathcal {R}_{2},\mathcal {R}_{3})$ are calibrated directly while ($\mathcal {R}_{2}$, $\mathcal {R}_{3}$ $\mathcal {R}_{1})$ are obtained by transfer

For both static and dynamic scene reconstruction, each Helmholtz camera was radiometrically calibrated as described in Sect. 5 using a calibration board with markers to define the position of the plane in each calibration shot. Position triangulation of any three of the four markers gives an anchor point and a normal to the calibration plane defined by the board. The black localisation markers are masked out together with the background outside the calibration boards in the images. Furthermore, data from more than just one pair of planes $\varPi _j$ and $\varPi _{j+1}$ is needed for continuous calibration coverage of the reconstruction frame. Using about 10 plane positions we directly calibrate for the $\mu$ distributions of three multi-spectral Helmholtz cameras $(\mathcal {R}_{1}, \mathcal {R}_{2},\mathcal {R}_{3})$ (a simplification of the more difficult problem of calibrating six single-frequency-spectrum Helmholtz cameras $(\mathcal {R}_{1,r}, \mathcal {R}_{1,g},\mathcal {R}_{2,b}, \mathcal {R}_{2,g}, \mathcal {R}_{3,r}, \mathcal {R}_{3,b})$ made possible by the assumption of identical R,G and B sensors in a single camera $\mathcal {C}$). Figure 15 shows the photometric parameter distributions in the region of interest for Monster used in the reciprocal constraint computation: the first column distributions are the directly calibrated distributions of $(\mathcal {R}_{1}, \mathcal {R}_{2},\mathcal {R}_{3})$ while the second column are the partner Helmholtz camera distribution obtained by transfer ($\mathcal {R}_{1}\rightarrow \mathcal {R}_{2}$, $\mathcal {R}_{2}\rightarrow \mathcal {R}_{3}$ and $\mathcal {R}_{3}\rightarrow \mathcal {R}_{1}$) from the directly calibrated (the transfer is essential for photometric parameter consistency within a reciprocal pair as described in Sect. 5). The maps are represented as heat maps to illustrate regional variation of $\mu$ within the scope of the reconstruction frame. The obtained photometric maps are invariant for all datasets acquired in the same capture session (static datasets in case of Fig. 15). An equivalent photometric calibration was performed for the dynamic scenes capture session.

Further, reconstruction of each shot is performed from three RGB images (three reciprocal pairs) instantaneously acquired by cameras $\mathcal {C}_1$, $\mathcal {C}_2$ and $\mathcal {C}_3$ under concurrent multispectral (RGB) illumination of $\mathcal {S}_1$, $\mathcal {S}_2$ and $\mathcal {S}_3$ as described in Sect. 7. Throughout the evaluation we, as appropriate, compare the following reconstruction methods:

1. VH: visual hull (i.e. shape-from-silhouette);
2. ML_HS_wPhCalib: standard (maximum likelihood) Helmholtz Stereopsis with photometric calibration only;
3. ML_HS_wPh&ChromCalib: standard (maximum likelihood) Helmholtz Stereopsis with photometric and chromaticity calibration;
4. BayesianHS_w/oCalib: uncalibrated Bayesian HS;
5. BayesianHS_wPhCalib: Bayesian HS with photometric calibration only;
6. BayesianHS_wPh&ChromCalib: Bayesian HS with photometric and chromaticity calibration.

We compare integration by Poisson surface reconstruction against the proposed final mesh assembly from the cloud of oriented vertices without explicit integration on static scenes. Subsequently, the explicit-integration-free pipeline is used throughout for all dynamic scenes.

**Fig. 16:**
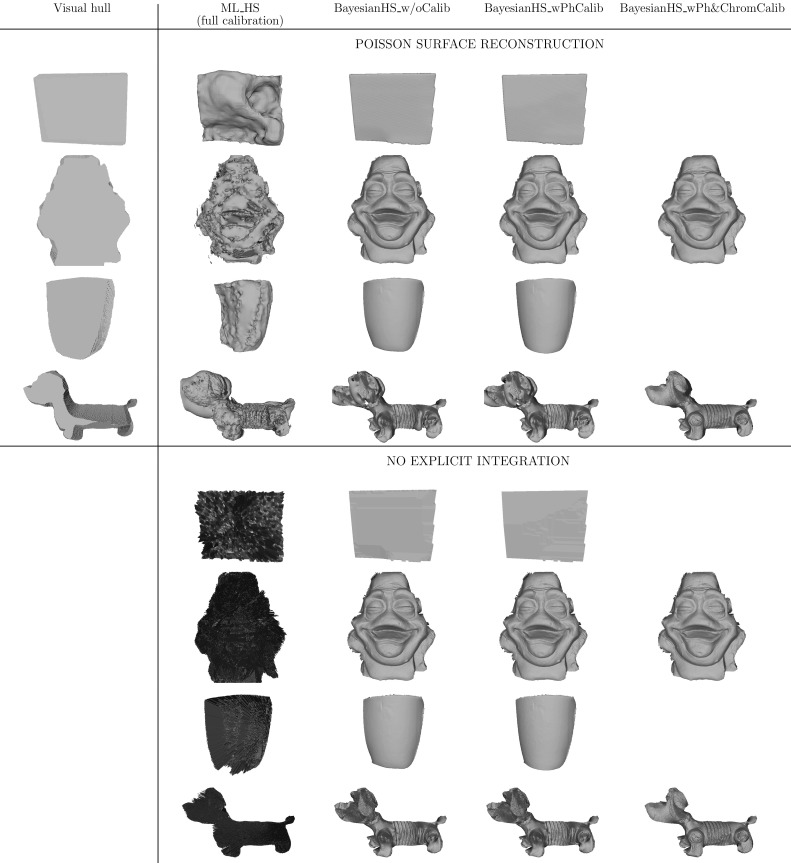
Static scene reconstruction with the fully calibrated ML HS and Bayesian HS at different levels of calibration, using Poisson Surface Reconstruction for final surface assembly and without explicit surface integration. The objects considered are: *Plane* resolution (spatially/depthwise): 3/0.5 mm; *Monster* initial resolution (spatially/depthwise)—1/0.5 mm; final resolution—0.25/0.03125 mm; *Mug* initial resolution (spatially/depthwise)—1/0.25 mm; final resolution—0.5/0.015625 mm; *Slinky* initial resolution (spatially/depthwise)—1/1 mm; final resolution—0.25/0.0625 mm; Visual hulls are also included for reference. All meshes are rendered in flat shading (without the use of per-vertex photometric normals)

### Static Scenes

This section is aimed at demonstrating both quantitatively and qualitatively the reconstruction accuracy of the proposed framework in static scene reconstruction of various photometric complexity.

#### Qualitative Evaluation

In Fig. 16 we qualitatively compare reconstructions of Plane, Monster, Mug and Slinky using

1. VH (all);
2. fully calibrated ML_HS: i.e. ML_HS_wPhCalib (Plane, Mug) or ML_HS_wPh&ChromCalib (Monster, Slinky);
3. BayesianHS_w/oCalib (all)
4. BayesianHS_wPhCalib (all)
5. BayesianHS_wPh&ChromCalib (Monster, Slinky)

Further, Poisson surface reconstruction is compared at different point cloud resolutions to direct meshing without explicit integration (the proposed alternative approach for generating a continuous surface from point clouds). Note that for Plane reconstructed with a single iteration of the coarse-to-fine framework Poisson surface reconstruction produces a much smoother continuous surface (used for quantitative evaluation in Sect. 8.1.3). For high resolution meshes of Monster, Mug and Slinky, there is either little difference visually between the two integration approaches (e.g. Monster, Mug, fully calibrated Slinky) or direct meshing performs better (e.g. under-calibrated Slinky) as the rim inaccuracies of the 2.5D reconstruction do not affect its performance as much as Poisson surface reconstruction. Based on the greater robustness observation and also being mindful of the known inherent risk of reconstructed point cloud alteration by an explicit integration method, direct meshing without explicit integration is consistently used in the remainder of this paper, instead of Poisson surface reconstruction, to obtain the final continuous surface from high-resolution point clouds.

**Fig. 17:**
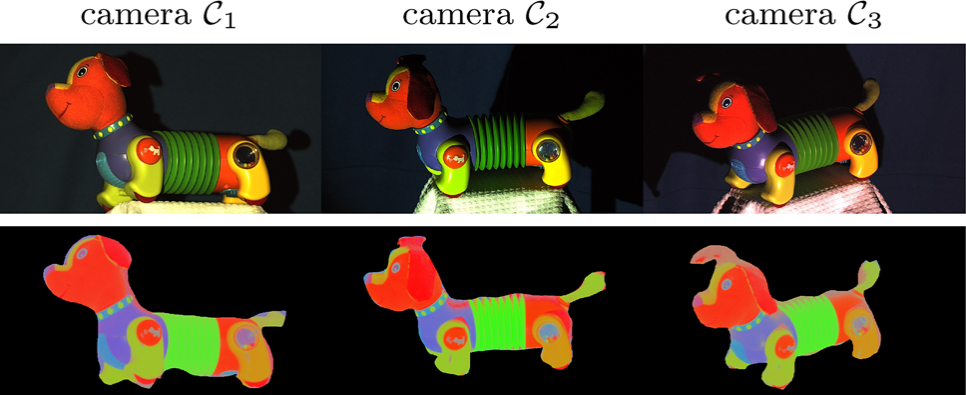
Spatial chromaticity estimation of Slinky for cameras $\mathcal {C}_{1}$, $\mathcal {C}_{2}$ and $\mathcal {C}_{3}$: intensity images showing pose and appearance under unfiltered projector illumination in the reference (calibration) frame (*top row*) and the estimated chromaticity maps (*bottom row*)

Let us make qualitative observations on the relative reconstruction method performance. The fully calibrated ML HS clearly fails on all four datasets regardless of the surface integration method chosen. As Plane and Mug are characterised by the system’s reference chromaticity (i.e. calibration object chromaticity), for these datasets the system is fully calibrated with just the photometric calibration. Some artefacts in the *uncalibrated* reconstructions (e.g. the bent inwards bottom-left-hand-side corner of Plane and the unnatural inflation of Mug) relative to the *calibrated* ones are already apparent visually. Full calibration for Monster and Slinky comprises both photometric and chromaticity parameter acquisition as their chromaticity is not the reference (or not uniformly so in case of Monster due to staining of plaster). The visual difference between Monster reconstructions by Bayesian HS at different calibration levels is subtle: a trained eye may notice the slight retraction of the lower lip of the fully calibrated result relative to the two under-calibrated ones. Analysis of such subtle differences is postponed to Sect. 8.1.3 where quantitative measurements against ground truth scans are presented. The role of chromaticity calibration is however more visible for the much larger local chromatic variations of Slinky. Figure 17 shows the surface chromaticity maps for Slinky obtained for each camera individually. They appear to be largely in agreement about the chromatic properties of the corresponding regions apart from some artefacts due to intensity sampling at grazing angles, sensor saturations and in the circular mirror region of the hind leg arguably having no inherent chromaticity. Chromaticity does not disambiguate between colours of different brightness and describes only their hue and saturation. Hence the estimated chromaticity will not match the colour of the object in the intensity images exactly as effectively whole colour families characterised by the same hue and saturation map onto the same chromaticity value. In addition, the definition of white spectrum in the system is the sum of RGB filter responses rather than the absolute white spectrum (approximated by unfiltered projector light). Full (photometric + chromaticity) calibration is key to getting a plausible result for Slinky. Photometric calibration is insufficient and is not even guaranteed to provide an incremental improvement on the completely uncalibrated result in case of non-reference chromaticity surface reconstruction as is clearly demonstrated by the fact that BayesianHS_wPhCalib performs as poorly as BayesianHS_w/oCalib. Recall that photometric calibration is reference dependent and unless it is used in conjunction with a chromaticity relative to the same reference resulting in the cancellation of the reference, the photometric calibration is not meaningful in the reconstruction of non-reference chromaticity surface. For Slinky the fully calibrated reconstruction is clearly superior showing a plausible global shape and a substantial degree of structural resolution on this highly challenging object. The dealt with challenges of Slinky include its signal scattering fine “furry” structure of the face, high frequency geometry in the accordion torso and the highly specular multi-coloured plastic of the rest of the body. BayesianHS_wPh&ChromCalib seems highly promising for multi-chromatic object reconstruction coping with substantial geometric and radiometric complexity.

#### Ground Truth

Let us describe the methods used to acquire the ground truth used in the quantitative evaluation (Sect. 8.1.3) on static data. Since Plane is the calibration board as in Fig. 8 in one of its orientations *not* used in photometric calibration, its ground truth can be easily computed using its markers as described in Sect. 5. The ground truth for Monster and Mug was obtained by laser scanning with the Creaform ZScanner 700CX (model VIUscan) whereas Slinky was captured using the structured light 3D scanning system David-SLS2. Due to the complex reflectance properties of Mug and Slinky neither scanning system was equipped to deal with, the surfaces had to be sprayed with talc powder to make them diffuse for ground truth acquisition.

**Fig. 18:**
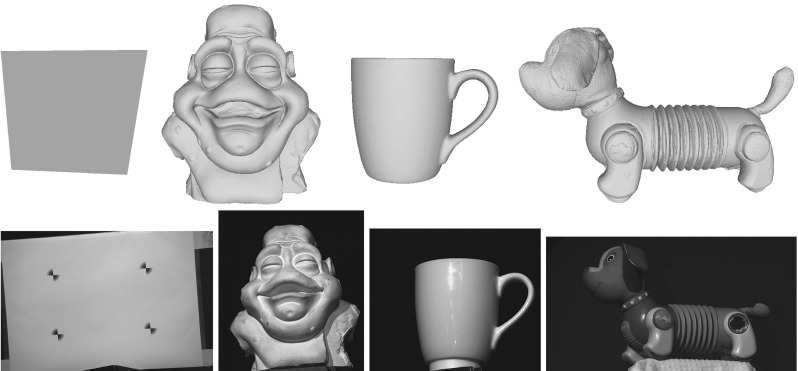
Ground truth and appearance under RGB illumination for static objects: Plane, Monster, Mug and Slinky

**Fig. 19:**
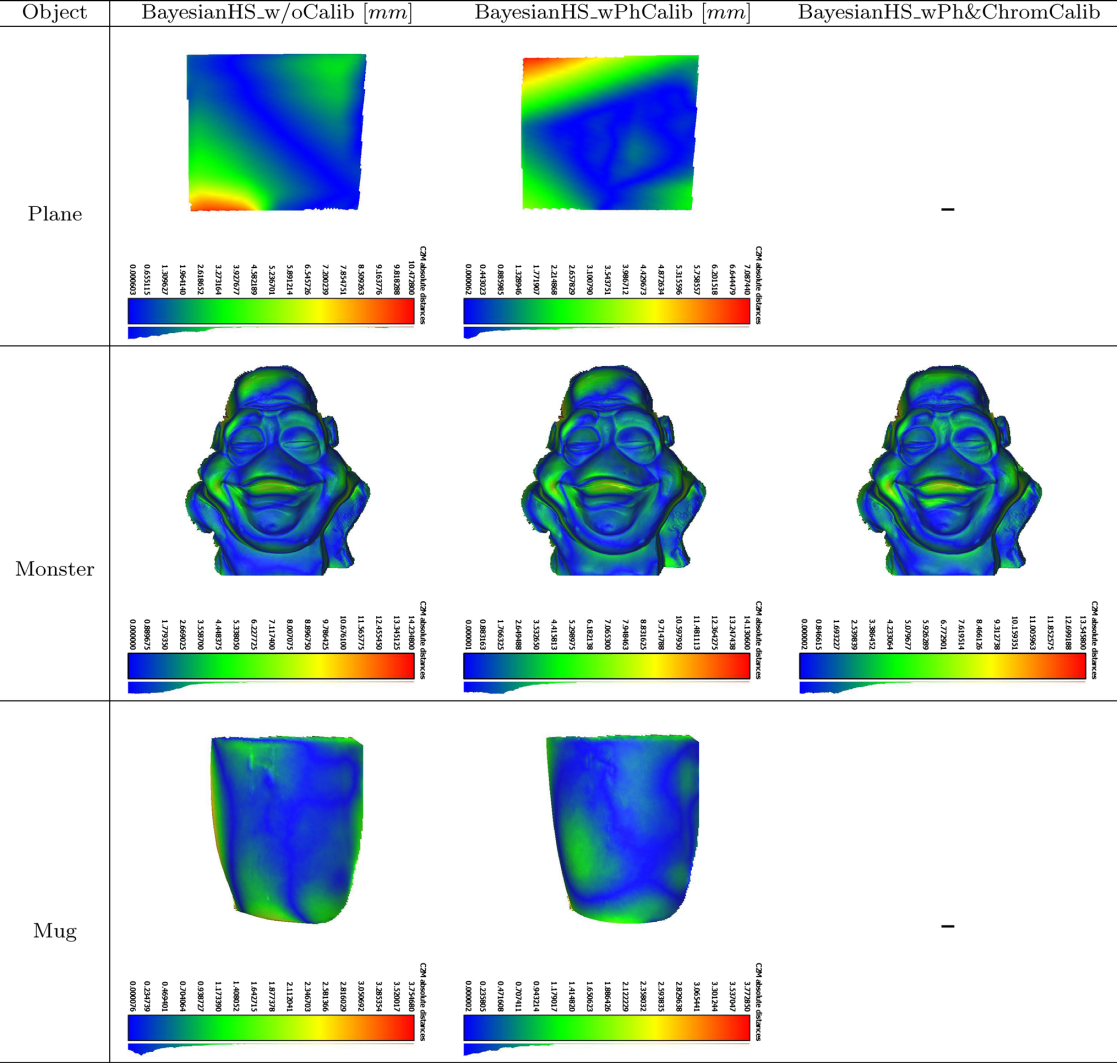
Spatial unsigned reconstruction error distribution of Bayesian CL HS at different calibration levels relative to the ground truth in Fig. 18

For the quantitative analysis, the models are aligned to the ground truth reference using ICP whenever possible preceded by coarse feature-based alignment. Since Slinky is only piecewise rigid, alignment of the model as a whole to the obtained ground truth is impossible as the object has deformed since the original capture. Only the rigid plastic front and back of the torso could potentially be aligned. Since model fragment alignment may be misleading, especially in the presence of global shape distorting artefacts on the outskirts of the 2.5D reconstructions, we do not quote any quantitative error metrics for Slinky. The ground truth meshes for static data is presented in Fig. 18.

**Table 2:** Global reconstruction error (rms and median) of the spatial distributions in Fig. 19 corresponding to Bayesian CL HS at different calibration levels

Object	BayesianHS_w/oCalib (mm)		Bayesian_wPhCalib (mm)		BayesianHS_wPh&ChromCalib (mm)	
Plane	rms	median	rms	median	rms	median
	3.06	1.34	2.24	1.18	–	–
Monster	rms	median	rms	median	rms	median
	2.48	1.53	2.58	1.71	2.50	1.62
Mug	rms	median	rms	median	rms	median
	0.80	0.38	0.58	0.34	–	–

#### Quantitative Evaluation

Quantitatively, the results are compared using the rms and median unsigned (model)vertex-to-(ground truth)mesh distance. These global quality metrics are presented in Table 2 for CL HS reconstructions of Plane, Monster and Mug from Fig. 16. The corresponding heat maps for the spatial error distributions are shown in Fig. 19. Note that the measurements for the particularly poor reconstructions by ML HS are omitted because, due to the difficulty in obtaining a satisfactory alignment for these meshes, any figures obtained would not be meaningful. As there can be no doubt of the inferiority of these methods from the qualitative comparison, the values are also not instrumental. Further, as described previously, Plane and Mug do not require chromaticity calibration being characterised by uniform reference chromaticity - photometric calibration suffices in this case and BayesianHS_wPh&ChromCalib is redundant.

The advantages of calibration are confirmed quantitatively by Plane and Mug datasets where Bayesian_wPhCalib shows both rms and median scores improved relative to BayesianHS_w/oCalib. The sub-millimetre rms errors on Mug are impressive considering the reflectance complexity of the object (see its appearance in Fig. 18).

For Monster partial (photometric only) calibration as expected deteriorates the performance. The fact that the scores of fully calibrated (BayesianHS_wPh&ChromCalib) and uncalibrated (BayesianHS_w/oCalib) algorithms are close on this object are to do with the retracted lip artefact of BayesianHS_wPh&ChromCalib clearly visible in the corresponding heat map in Fig. 19. The reason for the artefact is the extreme difficulty in accurate estimation of chromaticity inside the mouth and along the curvature of the lips due to self-occlusions and self-shadowing. Through MRF optimisation the effect of such chromaticity inaccuracies may be non-local i.e. a more global distortion such as the observed lip artefact. The positive observation is however that, for this object not showing drastic deviations from the reference chromaticity unlike Slinky, the uncalibrated algorithm is in fact robust enough to produce 2.5 mm accurate reconstructions in the absence of the exact chromaticity estimates and photometric calibration.

**Fig. 20:**
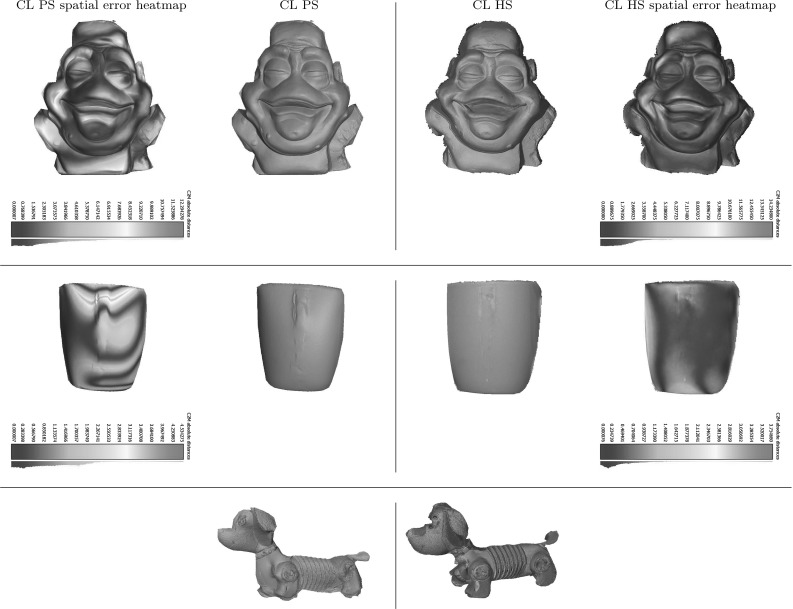
A comparison of Colour Photometric Stereo (CL PS) and Colour Helmholtz Stereopsis (CL HS) on static scenes: Monster, Mug: both CL PS and CL HS are uncalibrated; Slinky: CL PS with chromaticity compensation and fully calibrated CL HS

#### Comparison with Colour Photometric Stereo

In this section, the performance of standard Colour Photometric Stereo (CL PS) is compared against CL HS on static scenes involving Monster, Mug and Slinky. Standard CL PS is void of photometric or surface chromaticity calibration. Hence for fairness of experiment for Monster and Mug the comparison is against uncalibrated CL HS as well. Due to the extremely poor reconstruction quality of Slinky without chromaticity calibration, a comparison of uncalibrated methods would not be meaningful in this object’s case. Instead fully calibrated CL HS is compared against CL PS with a compensation for signal distortion due to surface chromaticity. The compensation, based on the same chromaticity map used in CL HS, involves division of the observed per-channel intensities at each pixel $[i_{c(r)}, i_{c(g)}, i_{c(b)}]$ by the corresponding component of the pixel’s chromaticity triplet $[p_{c,R}, p_{c,G}, p_{c,B}]$ where *c* is the camera used for CL PS. For quantitative evaluation (possible for Monster and Mug only) the ground truth in Fig. 18 is used. The scale of the photometric stereo results is adjusted to the ground truth during their alignment in order to be able to compare CL HS and CL PS in terms of world metric units.

CL HS clearly outperforms standard CL PS on Mug and Slinky (see Fig. 20). Due to its independence of the reflectance model CL HS copes with the specularities of Mug substantially better than CL PS while the superior accuracy of global shape is facilitated by the method’s dual characterisation of the surface by both depth and normals. Quantitatively, CL HS is better by just over 0.5 mm in its final rms scores (Table 3). Although neither CL PS nor Cl HS cope fully with the numerous heterogeneous complexities of Slinky, CL HS resolves structural detail better and is not prone to flatness of the global shape as much as CL PS. The virtual reconstruction camera of CL HS is not the same as the photometric stereo camera meaning that some areas within the scope of CL PS are difficult to reconstruct extremities for CL HS (e.g. Slinky’s chest).

Qualitatively, it is not clear which method performs better on Monster whose Lambertian reflectance model is exactly tailored to CL PS. The reconstruction by CL PS is smoother due to the normal integration as post-processing but visually somewhat inflated relative to CL PS. Quantitatively, the spatial error distributions and the global metrics once again indicate the superiority of CL HS.

### Dynamic Scenes

We have shown accurate geometric reconstruction of static scenes obtained from instantaneously acquired data, which indicates the great potential of CL HS for dynamic scene reconstruction. In this section, we evaluate the performance of the proposed method on dynamic scenes featuring non-rigid object deformation. The chosen datasets are geometrically complex and/or exhibit reflectance behaviour characterised by a non-Lambertian possibly spatially varying directional component of the reflectance model. Furthermore, the scenes are classified into three categories in order of increasing chromatic complexity: 1. uniform reference chromaticity; 2. uniform arbitrary chromaticity and 3. spatially varying chromaticity. Only the spatially varying chromaticity case requires the use of the entire scope of the pipeline in Fig. 6 whereas the other ones permit procedure simplifications. Along with the snapshots of the dynamic reconstruction results shown in this paper, as supplementary electronic material, we provide a video containing reconstruction results for full dynamic sequences (the video is also available for download at http://cvssp.org/projects/colourhs/video/).

#### Uniform Reference Chromaticity

**Table 3:** Quantitative comparison of CL PS and CL PS: global accuracy scores for some static scenes

Object	CL PS error (mm)		CL HS error (mm)	
Monster	rms	median	rms	median
	3.61	2.16	2.48	1.53
Mug	rms	median	rms	median
	1.37	0.98	0.80	0.38

**Fig. 21:**
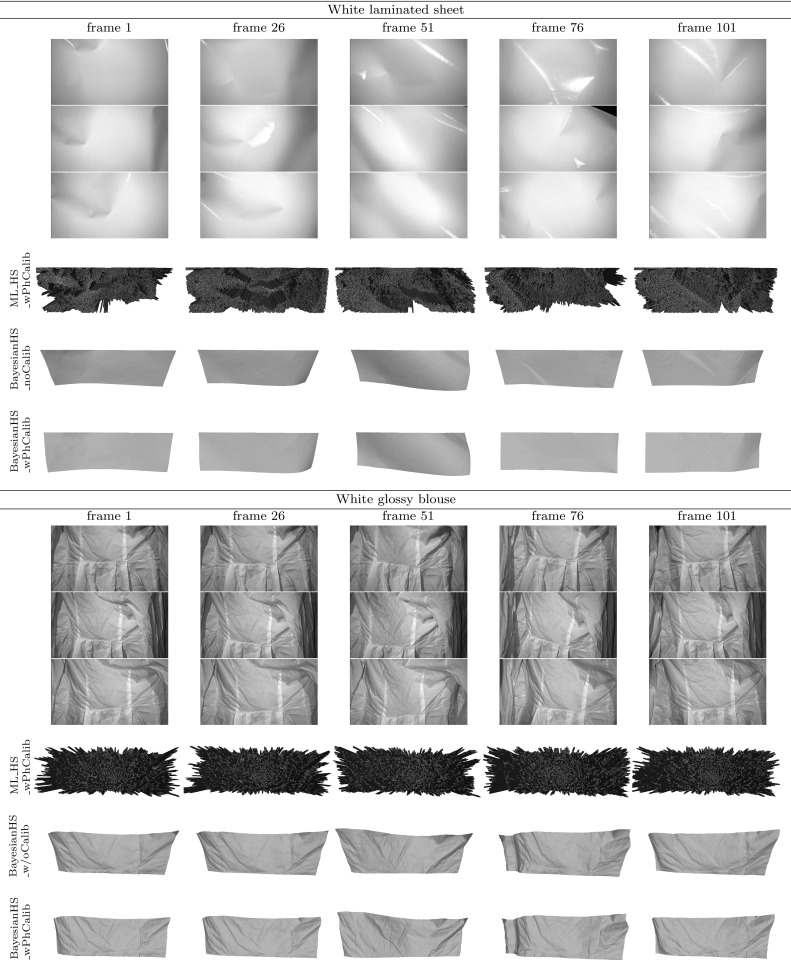
Reconstruction, uniform white chromaticity dynamic datasets: white laminated sheet and white glossy blouse. Input intensity images of $\mathcal {C}_1$, $\mathcal {C}_2$ and $\mathcal {C}_3$ and the reconstruction results using ML_HS_wPhCalib, BaysianHS_w/oCalib and BayesianHS_wPhCalib. Initial sampling resolution (spatially/depthwise): 3/0.5 mm. Final resolution 0.375/0.03125 mm. The presented meshes rendered in flat *shading* were assembled without explicit surface integration. Note that only a portion of the scene was reconstructed due to framing

Scenes with uniformly reference chromaticity do not require chromaticity calibration being fully calibrated for by the photometric calibration relative to the reference. In order to evaluate performance of CL HS on photometrically complex dynamic scenes independently of chromaticity calibration, we first present the results for this class of datasets. The two datasets chosen feature non-rigid deformation of a white highly specular laminated sheet (“WLS”) and a white glossy blouse (“Blouse”). The input video sequences of 201 frames are included in the supplementary material video together with the dynamic reconstruction results. Fig. 21 shows a sample set of five input frame sets (25 frames apart) from each sequence and the corresponding reconstructions by ML_HS_wPhCalib, BaysianHS_w/oCalib and BayesianHS_wPhCalib. Note that since the objects are of reference chromaticity like Plane and Mug in Sect. 8.1.3, the chromaticity calibration branch of the pipeline in Fig. 6 is not used.

**Fig. 22:**
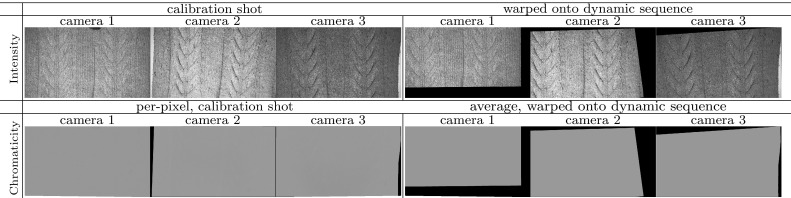
Chromaticity calibration with parameter averaging for Jumper. *Left column:* intensity images for cameras $\mathcal {C}_{1}$, $\mathcal {C}_{2}$ and $\mathcal {C}_{3}$ under unfiltered projector light and the corresponding per-pixel chromaticity maps for the reference frame. *Right column:* intensity alignment of the reference (calibration) frame to frame 1 of the dynamic sequence (see Fig. 23) initialising the region of interest for the reconstruction and the average-based chromaticity map per camera in the region of interest

The reflectance behaviour, particularly that of the laminated sheet, is complex with its pronounced specularities undoubtedly problematic for both conventional and photometric stereo. Bayesian HS as before clearly outperforms standard fully calibrated ML HS. Bayesian HS produces results with a remarkable level of detail resolution (see Blouse) and global accuracy. Due to the method’s reflectance model independence, even the most drastic non-Lambertian behaviour (e.g. the specularites of WLS) and unconstrained geometry are successfully coped with reproducing the folds, creases and domes of the deforming objects in the reconstructed meshes. Photometric calibration is also clearly essential for global accuracy of the reconstructed material patch as can be observed comparing the performance of Bayesian HS with and without photometric calibration on both Blouse and WLS. The characteristic global shape errors without photometric calibration include the somewhat retracted material patch orientation and the oddly enlarged and/or stretched prominent features. The uncalibrated method also seems to resolve the creases of Blouse less well. Upon close examination of the reconstruction videos (see the supplementary material), Bayesian HS shows similar temporal stability with and without calibration. Frame-by-frame comparison however reveals a consistent blurring of fine structural detail when the method is uncalibrated.

#### Uniform Arbitrary Chromaticity

If chromaticity is uniform but non-reference, relative photometric calibration is no longer tailored to the surface and hence chromaticity calibration becomes essential. However, if it is uniform or nearly so, an average estimate per camera can be used instead of per pixel calibration. For the class of uniform arbitrary chromaticity, fabric deformation of a woollen jumper (“Jumper”) has been reconstructed. Although the reflectance model in this case is simpler than in Sect. 8.2.1, the fabric is characterised by a much greater geometric complexity at the macro and the micro scales, respectively the plait structures and the actual criss-crossing of the knitwear thread. The dynamic sequence exhibits a sufficient amount of variation in the motion type having both fabric deformation and lateral translation.

**Fig. 23:**
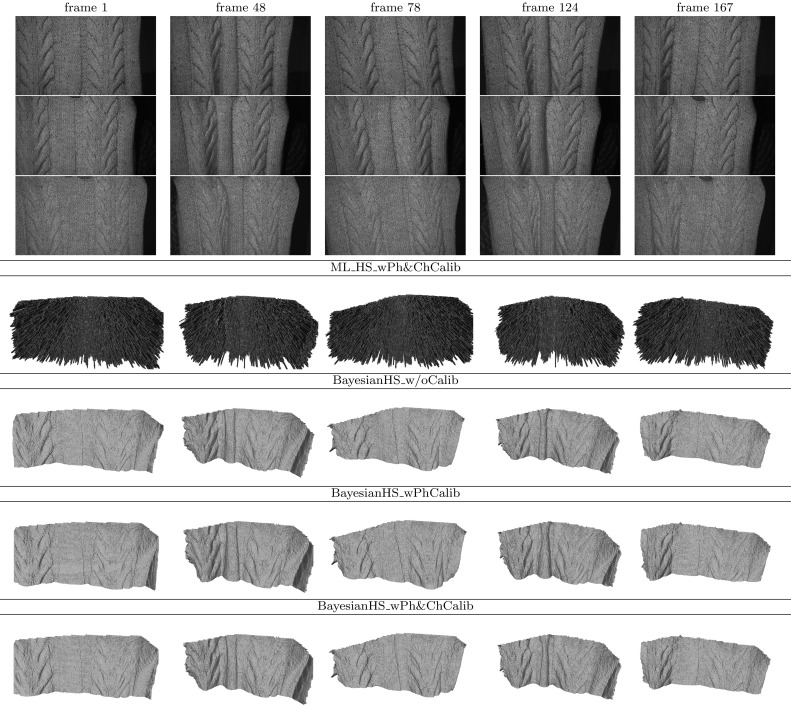
Reconstruction, uniform arbitrary chromaticity dynamic dataset with average-based chromaticity calibration: Jumper. Input intensity images of $\mathcal {C}_1$, $\mathcal {C}_2$ and $\mathcal {C}_3$ and the reconstruction results using ML_HS_wPh&ChCalib, BaysianHS_w/oCalib, BayesianHS_wPhCalib and BayesianHS_wPh&ChCalib. Initial sampling resolution (spatially/depthwise): 3/0.5 mm. Final resolution: 0.375/0.03125 mm. The presented meshes rendered in flat shading were assembled without explicit surface integration. Note that only the chromatically calibrated portion of the fabric was reconstructed

Figure 22 shows the reference frame of chromaticity calibration and the estimated per-pixel chromaticity maps for the captured jumper fabric. Subsequently, averaging is only performed over the homogeneous (i.e. woollen fabric) portions of the image but including inherent discolouration thereof (gaps in the knitwear, staining). In order to approximately align the calibrated region with the first frame of the dynamic sequence homography is performed for each camera. The reconstruction is limited to the overlapping region between the calibration reference frame and the dynamic sequence frame. The alignment quality of the reference frame to the dynamic sequence can be assessed by comparing the warped intensity image in Fig. 22 to the first frame of the dynamic sequence in Fig. 23. The overlapping area initialises the region of interest for the reconstruction throughout the entire sequence and is assigned the calibrated averaged chromaticity for each camera individually. Tracking is used to propagate the overlapping region to subsequent frames in the sequence hence providing a mask for the reconstruction. The average chromaticity may be applied to a larger reconstruction area of the fabric in case its uniformity in all images is a given. In this work we do not make that assumption and hence reconstruct only the chromatically calibrated by an average portion of the fabric. Average-based chromaticity maps per camera in the region of interest are also presented in Fig. 22.

Figure 23 shows a sample set of 5 frames from the Jumper sequence each reconstructed by ML_HS_wPh&ChCalib, BaysianHS_w/oCalib, BayesianHS_wPhCalib and BayesianHS_wPh&ChCalib. The full set of Jumper results with the input and the reconstructed sequences per method each featuring 200 frames can be found in the supplementary material video. From the sample set in Fig. 23 ML_HS_wPh&ChCalib, although retaining the basic trend line of the deformation, is clearly noisy and incapable of resolving any geometric detail. Without chromaticity calibration of Bayesian HS in the Jumper sequence, untailored relative photometric calibration introduces abnormal feature-inflating instability. Chromaticity calibration definitely moderates the feature inflation of the partially calibrated result settling in-between the uncalibrated and partially calibrated results. Without ground truth it is not obvious whether the fully calibrated or the uncalibrated reconstructed sequence is more accurate as both look plausible. The accuracy of the uncalibrated sequence depends on the validity of the assumption that the photometric characteristics of the Helmholtz cameras are near-identical in the region of interest and the chromaticity is largely characterised by an equal response on all channels. The accuracy of the fully calibrated result depends on how representative the average chromaticity is of the entire surface of the fabric for the overall calibration balance with the relative photometric calibration. Figure 22, showing the per-pixel chromaticity maps for each viewpoint, exposes non-uniformities in the chromaticity distribution. However, these are brought about by the knitwear translucency as well as the transparencies through the gaps in the woven fabric and should best be ignored. Since these non-uniformities contribute to the average it is but an approximation of the true uniform chromaticity of Jumper. Nonetheless, the assumption of the fully calibrated reconstruction pipeline is more likely, instilling more confidence in the accuracy of its result.

**Fig. 24:**
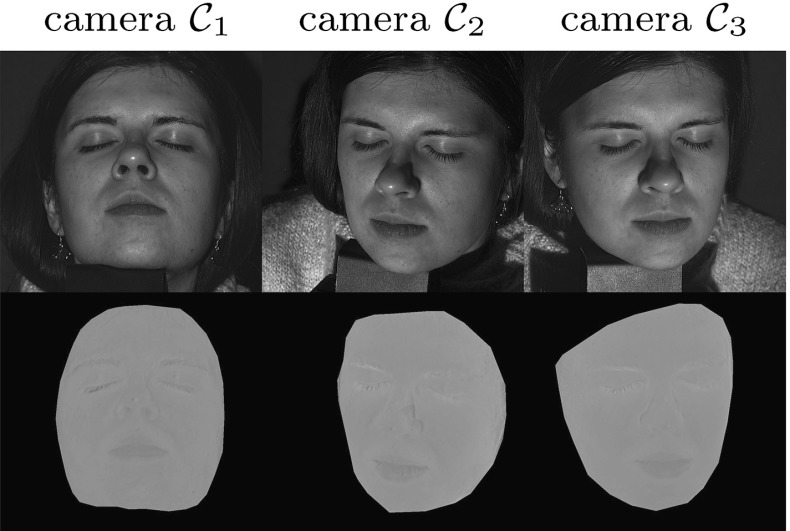
Spatial chromaticity estimation of Face for $\mathcal {C}_1$, $\mathcal {C}_2$ and $\mathcal {C}_3$: intensity images showing pose and appearance under unfiltered projector illumination in the reference (calibration) frame (*top row*) and the estimated chromaticity maps (*bottom row*)

We have shown that, given approximately uniform chromaticity, material chromaticity averaging per camera still permits reconstruction. The possibility for chromaticity calibration simplification is useful particularly if per-pixel alignment and tracking is difficult as is the case with the intricate structure of Jumper’s knitwear.

**Fig. 25:**
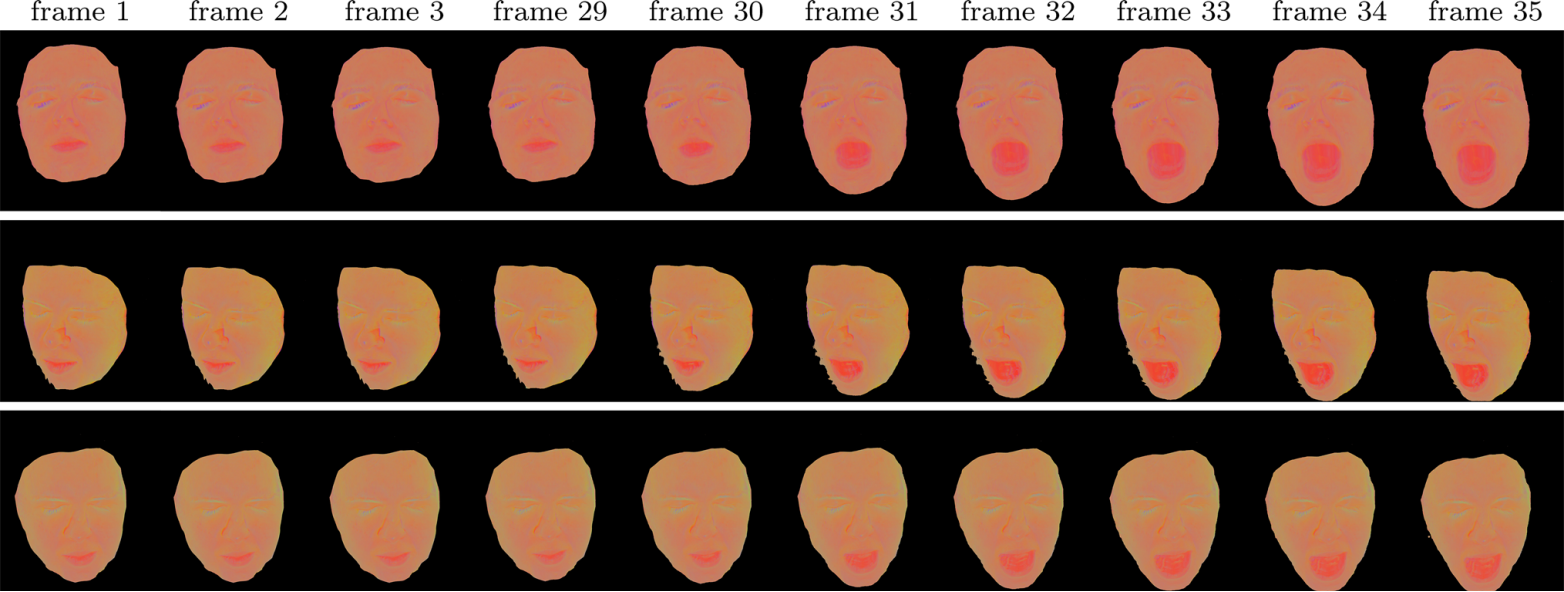
Temporal chromaticity propagation within the dynamic sequence face for $\mathcal {C}_1$ (*top*), $\mathcal {C}_2$ (*middle*) and $\mathcal {C}_3$ (*bottom*)

#### Spatially Varying Chromaticity

Dynamic scenes with spatially varying chromaticity are the most complex case for CL HS requiring full utilisation of the processing pipeline without simplification of calibration. We evaluate the performance in this case on a dynamic sequence with human facial expressions (“Face”), which poses several reconstruction challenges. Firstly, a sufficient degree of spatial chromaticity variation is present due to facial features (lips, eyebrows etc.) and natural skin imperfections as no make-up was used in the capture to even out the skin tone. Secondly, the face has a non-Lambertian reflectance model with specular highlights. Thirdly, the dynamic sequence offers a wide range of geometrically complex fast changing grimaces to thoroughly test both per-frame accuracy and temporal consistency of the non-rigid deformation reconstruction.

**Fig. 26:**
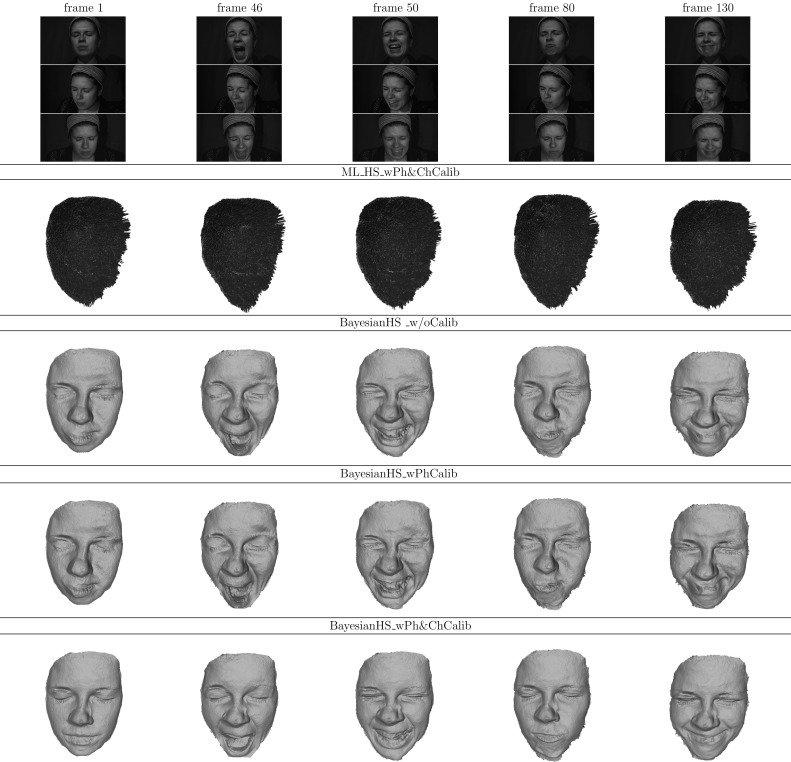
Reconstruction, spatially varying chromaticity dynamic dataset: Face. Input intensity images of $\mathcal {C}_1$, $\mathcal {C}_2$ and $\mathcal {C}_3$ and the reconstruction results using ML_HS_wPh&ChromCalib, BaysianHS_w/oCalib, BayesianHS_wPhCalib and BayesianHS_wPh&ChromCalib. Initial sampling resolution (spatially/depthwise): 1/0.5 mm. Final sampling resolution: 0.375/0.03125 mm. The presented meshes rendered in flat shading were assembled without explicit surface integration

Figure 24 shows the appearance of the face in the calibration reference frame of each camera and the corresponding estimated per-pixel chromaticity map that is essential for accurate reconstruction given spatially varying chromaticity. Each chromaticity map captures the spatial skin chromaticity variation at the scale ranging from facial features to local hyper-pigmentation. Skin chromaticity estimation is inherently difficult due to sub-surface scattering violating the assumption of a topical reflectance model (BRDF). Since sub-surface scattering is directionally variant, it is not surprising that per-camera chromaticity estimates in Fig. 24 differ from each other.

The calibration pose in Fig. 24 is significantly different from the pose in the first frame of the dynamic sequence (see Fig. 26). The result of aligning the reference chromaticity maps to the first dynamic sequence frame by warping as described in Sect. 6.2 are presented in Fig. 25. The figure also shows optical-flow-based propagation of the aligned chromaticity maps through the sequence. Optical flow tracking shows stability with the chromaticity maps staying constant in the initial static part of the sequence. At the same time during the part of rapid motion from frame 29, the chromaticity maps evolve accordingly with the chromaticity estimates propagated to the new shots. The interior of the mouth unseen in the reference (calibration) frame acquires the colour of the chromatically similar lips, which is not a bad guess for the unavailable information. Propagation of chromatically distinct regions relative to each other is consistent as optical flow errors tend to occur mainly within chromatically uniform areas.

Figure 26 compares reconstruction results using ML_HS_wPh&ChromCalib, BaysianHS_w/oCalib, BayesianHS_wPhCalib and BayesianHS_wPh&ChromCalib. As in previously presented results, fully calibrated ML HS is still absolutely no match for Bayesian HS. BayesianHS_wPhCalib with a partial (photometric only) calibration does not offer any apparent benefits over uncalibrated Bayesian HS as both show severe global shape distortions. Untailored photometric calibration in the absence of chromaticity calibration makes the algorithm more sensitive to noise with a decrease in smoothness on the cheeks. Incomplete (as well as lack of) calibration also causes a global distortion of photometric normal orientation. Although not flawless, the reconstructions obtained with the fully calibrated pipeline are very good considering that the reconstructed surface exhibits sub-surface scattering violating the assumptions of both the chromaticity estimation procedure and Helmholtz Stereopsis in general. Note how the fully calibrated Bayesian HS copes with the specular highlights on the cheeks. The algorithm even correctly reconstructs the teeth that are the uncalibrated elements of the scene, exposed in the course of the scene playing out. The teeth are characterised by a specular reflectance model and a chromaticity with an approximately equal inter-channel relationship. On the other hand, self-occlusions (e.g. by the nose) create difficult to reconstruct shadowed regions of low intensity and result in artefacts such as the bump on the right-hand side of the nose. Such self-shadowing is a common problem with acquisition set-ups consisting of a limited number of cameras.

A video showing 200 frames of the dynamic Face sequence reconstruction by all compared methods is supplied in the supplementary material. The fully calibrated Bayesian HS remains plausible throughout the sequence. The slight deterioration in the lip reconstruction at the end of the sequence is caused by the washing out of the region boundaries in the propagated chromaticity map towards the end of the sequence due to drift (i.e. numerical error accumulation). The observation is typical for tracking over long sequences.

## Conclusion and Future Work

In this paper we have proposed Colour Helmholtz Stereopsis (CL HS) - a novel multi-spectral framework for Helmholtz Stereopsis (HS) suitable for reconstruction of dynamic scenes with complex unknown spatially varying BRDFs. This significantly improves upon conventional and photometric stereo which are inherently limited to surfaces with simple or known reflectance models, but also upon standard HS which has till now been limited to static scenes. Incorporation of wavelength multiplexing into HS for the first time enables simultaneous acquisition of the minimum number of reciprocal image pairs required for dynamic scene reconstruction but also introduces an additional set of challenges due to sampling the BRDF of the surface at different wavelengths.

To address the challenges, in this paper we decompose the BRDF into a directional component dependent only on the sampling geometry (i.e. the illumination incidence and viewing angles) and the chromaticity component. The arbitrariness of the directional component is inherently guaranteed by the core reciprocity principle of HS. In order to enable an equally arbitrary spatially varying chromaticity component, we have proposed a novel spatio-temporal chromaticity calibration procedure. The procedure consists of spatial chromaticity estimation performed on a static reference frame and the subsequent temporal chromaticity propagation based on dense tracking by optical flow to transfer the estimated parameters to any unseen frame given a sufficient overlap with the reference. Further, the chromaticity estimation procedure complements our practical photometric Helmholtz camera calibration, which addresses the problem of inter-channel signal consistency in the multi-spectral acquisition set-up. Specifically, the proposed photometric and chromaticity calibration procedures are tailored to each other by being both relative to the same reference chromaticity, which cancels out resulting in a universal reconstruction pipeline. Lastly, we have implemented Bayesian HS in order to eliminate the computational inadequacies of standard maximum likelihood (ML) HS in reconstruction with a minimal input of three reciprocal pairs per shot characteristic of CL HS.

The proposed fully calibrated CL HS pipeline has been validated quantitatively and qualitatively on both static and dynamic real scenes of varying structural and photometric complexity. Using CL HS we have obtained high quality results on dynamic deformation sequences of highly non-Lambertian surfaces fundamentally challenging for the established techniques. The obvious comparison of CL HS against the established Colour Photometric Stereo (CL PS) on static scenes for ease of quantitative comparison has been presented to illustrate the latter’s limitations in tackling complex reflectance and geometry. CL PS has been shown to have an inferior performance to CL HS by up to over 1 mm higher rms error, quantitatively, and, qualitatively, by an obvious shape distortion, both globally and locally. The performance of CL HS on chromatically different scenes, ranging from uniform white to spatially varying, validates the wide applicability and accuracy of the method. The necessity of the proposed full calibration procedure for arbitrary chromaticity surfaces has been validated in a comparative evaluation against its uncalibrated and partially calibrated pipeline variants. Quantitatively, the reconstruction accuracy of CL HS measured as an rms error value on versatile static scenes with ground truth is in the range of 0.5–2.5 mm (the corresponding median error range is 0.3–1.7 mm) with the smooth untextured highly specular object (Mug) showing the greatest accuracy. A comparison to the standard ML HS formulation has left no doubt of the superiority of the proposed choice of the Bayesian formulation. As the result after the extensive validation, we can conclude that the CL HS framework presented in this paper is the first widely applicable method with a practical set-up of just three camera/light source pairs that is capable of reconstructing dynamic scenes with arbitrary spatially varying reflectance i.e. with equally unconstrained directional and chromaticity BRDF components.

In the paper several sources of error in our complex multi-spectral system have been highlighted. While some (e.g. cross-talk) have not been found dominant, others inspire future work. One direction for future work could be an improvement of the tracking results for chromaticity propagation. At the moment, drift accumulated when tracking over longer sequences distorts chromaticity maps which negatively affects reconstruction by corruption of the HS constraint formulation and consequently the depth/normal estimates. Along with the obvious periodic chromaticity re-estimation throughout the sequence, recent approaches based on non-sequential tracking (Klaudiny et al. 2012; Budd et al. 2013) could be used to reduce drift. Although adding additional computational complexity, such methods could help address, for example, the observed issue of region boundary resolution loss in the propagated chromaticity maps.

Another avenue for future work would be to investigate how the method could be made robust to specular highlights. Although HS is unaffected by specular reflectance models provided the camera sensor is not saturated, specular highlights will impair tracking which is key in accurate chromaticity propagation. The proposed framework has been shown to cope with localised specularities (see the Face dataset) but it will cause problems if larger areas are affected. The problem of ubiquitous specular highlights remains an open problem for the proposed system requiring a custom tracking approach, not relying on intensity matching, in order to prevent tracking failure. The additional tracking robustness could be provided by involving the estimated prior geometry in the tracking constraint. Unfortunately, however, saturations also cause errors in spatial chromaticity estimation introducing pseudo surface colours already in the initial state of the spatio-temporal chromaticity calibration procedure. If choosing a different calibration frame is not possible, such regions affected by extreme specular reflection could be removed prior to chromaticity estimation. These regions can be subsequently filled in based on chromaticity of the surrounding areas.

## Research Data Access

The authors confirm that the datasets generated as part of this research are freely available under the terms and conditions detailed in the licence agreement enclosed in the data repository. Details of the data and how to obtain access are available from the University of Surrey: doi:10.15126/surreydata.00811986 and the data repository website: http://cvssp.org/data/colourhs/.

## Electronic supplementary material

Below is the link to the electronic supplementary material.

Supplementary material 1 (wmv 68287 KB)
